# ATP Release from Chemotherapy-Treated Dying Leukemia Cells Elicits an Immune Suppressive Effect by Increasing Regulatory T Cells and Tolerogenic Dendritic Cells

**DOI:** 10.3389/fimmu.2017.01918

**Published:** 2017-12-22

**Authors:** Mariangela Lecciso, Darina Ocadlikova, Sabina Sangaletti, Sara Trabanelli, Elena De Marchi, Elisa Orioli, Anna Pegoraro, Paola Portararo, Camilla Jandus, Andrea Bontadini, Annarita Redavid, Valentina Salvestrini, Pedro Romero, Mario P. Colombo, Francesco Di Virgilio, Michele Cavo, Elena Adinolfi, Antonio Curti

**Affiliations:** ^1^Department of Experimental, Diagnostic and Specialty Medicine, Institute of Hematology L. and A. Seràgnoli, S. Orsola-Malpighi Hospital, University of Bologna, Bologna, Italy; ^2^Istituto Nazionale dei Tumori (IRCCS), Milan, Italy; ^3^Ludwig Cancer Research Center, Faculty of Biology and Medicine, University of Lausanne, Lausanne, Switzerland; ^4^Department of Morphology, Surgery and Experimental Medicine, University of Ferrara, Ferrara, Italy; ^5^Immunohematology Service and Blood Bank, Policlinico S.Orsola Malpighi, Bologna, Italy

**Keywords:** acute myeloid leukemia, chemotherapy, ATP, dendritic cell, T regulatory cells, immunosuppression

## Abstract

Chemotherapy-induced immunogenic cell death can favor dendritic cell (DC) cross-priming of tumor-associated antigens for T cell activation thanks to the release of damage-associated molecular patterns, including ATP. Here, we tested the hypothesis that in acute myeloid leukemia (AML), ATP release, along with its well-known immune stimulatory effect, may also contribute to the generation of an immune suppressive microenvironment. In a cohort of AML patients, undergoing combined daunorubicin and cytarabine chemotherapy, a population of T regulatory cells (Tregs) with suppressive phenotype, expressing the immune checkpoint programmed cell death protein 1 (PD-1), was significantly increased. Moving from these results, initial *in vitro* data showed that daunorubicin was more effective than cytarabine in modulating DC function toward Tregs induction and such difference was correlated with the higher capacity of daunorubicin to induce ATP release from treated AML cells. DCs cultured with daunorubicin-treated AML cells upregulated indoleamine 2,3-dioxygenase 1 (IDO1), which induced anti-leukemia Tregs. These data were confirmed *in vivo* as daunorubicin-treated mice show an increase in extracellular ATP levels with increased number of Tregs, expressing PD-1 and IDO1^+^CD39^+^ DCs. Notably, daunorubicin failed to induce Tregs and tolerogenic DCs in mice lacking the ATP receptor P2X7. Our data indicate that ATP release from chemotherapy-treated dying cells contributes to create an immune suppressive microenvironment in AML.

## Introduction

The cancer cell death induced by some chemotherapeutic agents, especially anthracyclines, such as daunorubicin (DNR), stimulates an effective antitumor T-cell immune response in solid tumors (1–3) and leukemias (1), including acute myeloid leukemia (AML). Such a death process, named immunogenic cell death, is characterized by intracellular modifications as well as alterations of tumor microenvironment, which elicit antitumor immune response and may account, at least in part, for the therapeutic effect of antineoplastic drugs (4). Among the different mechanisms underlying immunogenic cell death, autophagy-dependent extracellular release of ATP from chemotherapy-treated dying tumor cells is a key priming factor (5). Indeed, ATP, acting at the purinergic P2X7 receptor (P2X7R), drives dendritic cells (DCs) activation, thereby enabling the cross-presentation mode (5, 6).

**Graphical Abstract:**
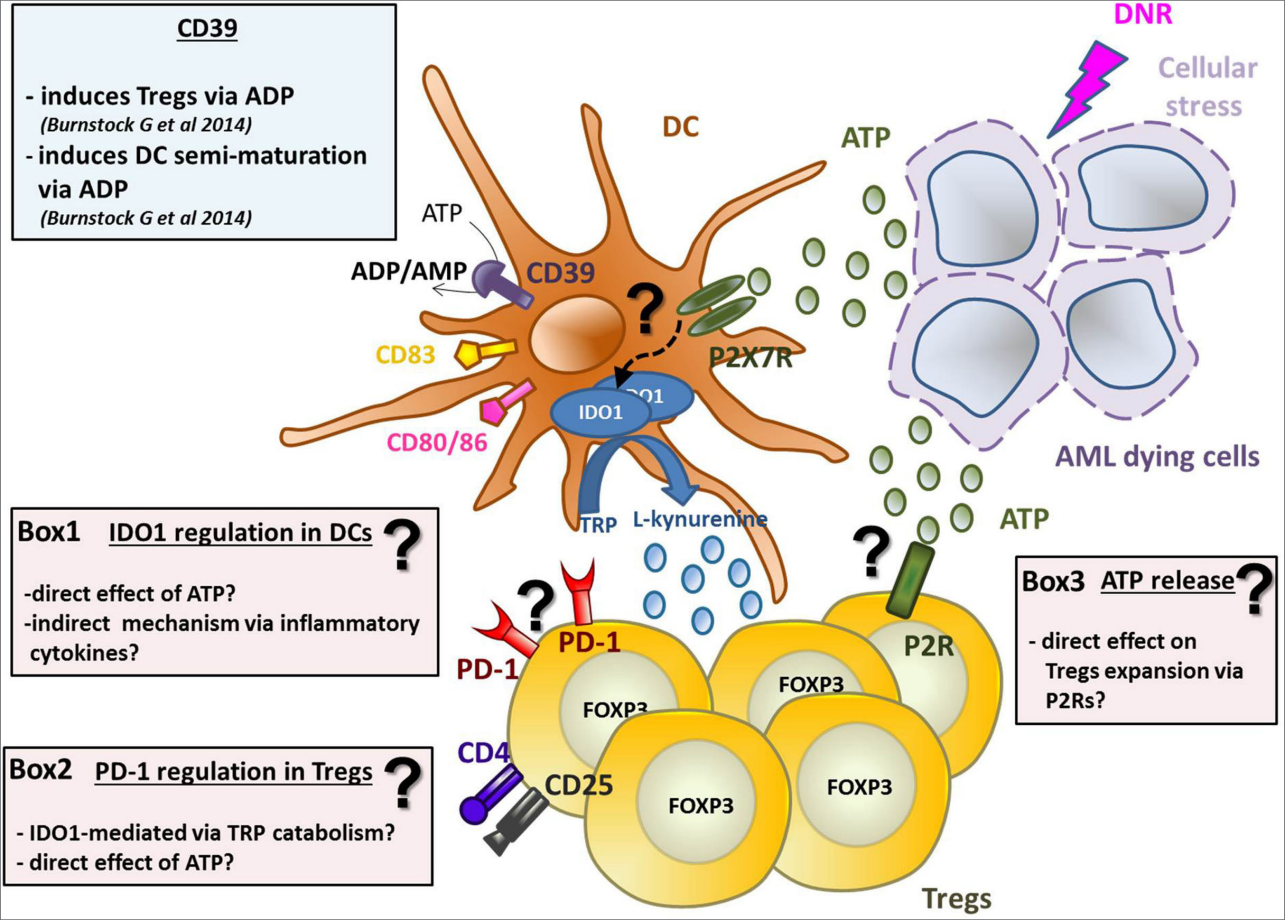
ATP released from chemotherapy-treated dying leukemia cells has tolerogenic effects by inducing IDO1-expressing DCs and increasing Tregs. DNR-treated AML dying cells release ATP, which induces DCs activation and maturation (in particular CD80, CD83, and CD86 upregulation) and IDO1 expression *via* purinergic P2X7 receptor. The mechanism of IDO1 upregulation is still unknown (see Box 1 for hypotheses). IDO1 catabolizes the conversion of tryptophan (TRP) into l-kynurenine inducing Tregs. Along with DCs maturation, ATP induces the upregulation of CD39, which converts ATP into ADP/AMP, known to induce semi-maturation of DCs and partial Th1 polarization of CD4^+^ T cells. On the other hand, AMP is known to impair maturation of DCs, thus decreasing the capacity of human DCs to prime CD8^+^ T cells leading to tolerance. ATP released from dying AML cells has two distinct effects on Tregs: (1) it induces their expansion and (2) PD-1 upregulation. The exact mechanisms underlying the effect of ATP on Tregs are still unclear (see Boxes 2 and 3).

More recently, some antineoplastic agents have been also associated with the generation of an immunosuppressive, rather than immunostimulant, tumor microenvironment (7–9), but the underlying mechanisms are still unknown. In particular, to our knowledge, a tolerogenic effect of ATP release from chemotherapy-treated dying tumor cells was never investigated in AML.

Acute myeloid leukemia cells have been shown to induce a suppressive microenvironment by expanding T regulatory cells (Tregs), which in turn may hamper anti-leukemia immune response (10). Although the direct activity of ATP on Tregs is well established (11–14), the contribution of ATP release from chemotherapy-treated AML cells on Tregs induction was never investigated. ATP and, more in general, inflammatory stimuli can stimulate DCs either to promote or suppress T-cell responses (15), the latter occurring through the generation of Tregs. The most relevant mechanism by which DCs induce Tregs is through the upregulation of indoleamine 2,3-dioxygenase 1 (IDO1) (15–18), an enzyme that degrades the essential amino acid tryptophan into kynurenine and is involved in the generation of an immunosuppressive microenvironment in AML (19, 20). Whether upon chemotherapy, along with its capacity of promoting DC-mediated cross-priming to tumor antigen-specific T cells, ATP may be implicated in conferring tolerogenic features to infiltrating DCs *via* IDO1 upregulation has not been specifically addressed.

In the present study, by moving from *ex vivo* analysis of T-cell composition emerging in AML patients after induction chemotherapy, we *in vitro* and *in vivo* investigated the effect of ATP release from chemotherapy-treated dying leukemia cells on the induction of an immune suppressive microenvironment in AML. In particular, we addressed the effect of ATP release from chemotherapy-treated AML cells on Tregs and DCs.

## Materials and Methods

### Cells

All human samples were collected from healthy donors (HD) and from newly diagnosed AML patients after informed consent (local Ethical Committee approval code: 147/2013/O/Tess). Patients’ characteristics are reported in Table S1 in Supplementary Material. AML cells were obtained as mononuclear cells isolated by Ficoll-Hypaque centrifugation (Amersham, USA) from patients’ bone marrow or peripheral blood (PB) samples, including at least 70% leukemic cells, as evaluated by morphology and FACS analysis. CD3^+^, CD19^+^, CD14^+^, and CD4^+^CD25^+^CD127^dim/−^ cells were purified by magnetic separation (Miltenyi Biotec, Germany), according to manufacturer’s instructions from mononuclear cells separated from buffy coats and patients’ PB by Ficoll-Hypaque centrifugation (Amersham). Purity of cell populations was always >90%. Human HL-60 (DMSZ; ACC 3, FAB M2) and murine WEHI-3B (DMSZ; no. ACC 26) AML cell lines were maintained at 37°C and 5% CO_2_. HL-60 cells were cultured in RPMI 1640 medium (Lonza, Milan, Italy), supplemented with 10% heat-inactivated fetal bovine serum (Gibco-Invitrogen, USA), 2 mM l-glutamine, 100 U/ml penicillin, and 100 µg/ml streptomycin (MP Biomedicals, Italy) (complete RPMI). WEHI-3B cells were cultured in Iscove modified Dulbecco’s medium (Sigma-Aldrich, USA) supplemented with 10% fetal bovine serum (Euroclone, Italy), 100 U/ml penicillin and 100 mg/ml streptomycin (Euroclone). AML cells (1 × 10^6^/ml) were treated with DNR 500 ng/ml (Sigma-Aldrich) or cytarabine (ARA-C) 25 µg/ml (Sigma-Aldrich) for 4 h and tested for apoptosis by Annexin-V-FLUOS Apoptosis Detection Kit (Roche, Switzerland) and ATP release (see Datasheet S1 in Supplementary Material).

### *Ex Vivo* Characterization of Leukemia-Reactive T Cells

T cells from newly diagnosed AML patients (*n* = 23) undergoing standard “7 + 3” induction chemotherapy regimen, including continuous infusion of ARA-C at 200 mg/m^2^ for 7 days and DNR at 60 mg/m^2^ for 3 days, were analyzed before drug administration and at different time points (+7, +14, +21, and +28) after the end of administration regimen. Due to paucity of evaluable cells, in few cases (*n* = 3), day 7 T cells were not analyzed. For IFN-γ intracellular detection, purified CD3^+^ T cells were co-cultured with autologous AML blasts, at 10:1 ratio, in complete RPMI supplemented with 10% autologous serum (autologous RPMI). CD3^+^ T cells either used as such or pre-activated with ionomycin (500 ng/ml; Sigma-Aldrich) and phorbol-12-myristate-13-acetate (PMA, 10 ng/ml; Sigma-Aldrich) and co-cultured with autologous CD19^+^ cells were used as controls. After 4 h, brefeldin A (2 µg/ml; BD Biosciences, USA) was added, followed by overnight incubation before permeabilization and staining.

In selected cases, 5 × 10^6^ CD3^+^ T cells from IFN-γ-responding AML patients were collected after chemotherapy and stimulated overnight with autologous AML blasts (ratio 1:1). Cells were incubated with IFN-γ and TNF-α Catch reagent (Miltenyi) for 5 min on ice. Finally, after incubation with medium for 45 min at 37°C, cells were stained with the following human monoclonal antibodies (mAbs): IFN-γ APC, TNF-α APC (Miltenyi), CD137 PE-Cy7 (4-1BB, 4B4-1; Biolegend, USA), CD3 APC-eFluor780 (SK7; eBioscience, USA), CD8 Pacific Blue (B9.11; BD Pharmingen, USA), CD33 FITC (HIM3-4), or CD34 FITC (561) (both from Biolegend, USA) to discard blasts population from analysis. CD3^+^CD8^+^IFN-γ^+^TNF-α^+^CD137^+^ were sorted at BD FACS Aria cell sorter (BD Biosciences) and *in vitro* expanded (2–3 weeks) on a irradiated mononuclear cell feeder layer from two HD (1 × 10^6^ cells/ml) in RPMI supplemented with human serum (HS, 8%), phytohemagglutinin (PHA, 1 µg/ml; Sigma-Aldrich) and IL-2 (150 U/ml; Roche), for 2–3 weeks. IL-2 was added to the culture every other day.

### DC Generation, Maturation, and Pulsing

Human monocyte-derived DCs were generated by a 5-day culture of CD14^+^ cells in complete RPMI in presence of granulocyte-macrophage colony stimulation factor (50 ng/ml; GM-CSF Endogen, USA) and IL-4 (800 U/ml; Miltenyi), as previously described (21, 22). DC maturation was induced with a cocktail of cytokine made of TNF-α (10 ng/ml; Endogen), IL-6 (10 ng/ml; Endogen), IL-1β (10 ng/ml; Endogen), and 1 µg/ml PGE_2_ (Endogen) (23) or with ATP (1 mM; Sigma-Aldrich). For DC pulsing, chemotherapy-treated HL-60 cells were cultured for 20 h with immature DCs (2:1 ratio) in autologous RPMI. After culture, IDO1 protein expression was evaluated by Western blotting (see Datasheet S1 in Supplementary Material).

### *In Vitro* Induction of IFN-γ-Producing Leukemia-Specific T Cells

Leukemia-reactive IFN-γ producing CD3^+^ T cells were evaluated after two rounds of *in vitro* stimulation (7 + 7 days) with autologous DCs pulsed with chemotherapy-treated HL-60 cells (ratio 10:1) in autologous RPMI. IL-2 (20 U/ml) was added on alternate days. In selected experiments, allogeneic Tregs induced by the same DCs used for T cell priming were added to cell cultures (ratio 1:1) for 5 days to test their suppressive activity. At the end of culture, T cells were tested for IFN-γ production. DCs loaded for 24 h (1:2 ratio) with HL-60 cell lysate, obtained after three cycles of cells freeze-thawing and filtering through an insulin syringe, were used as target.

Antigen-specific IFN-γ-producing CD3^+^ T cells were obtained as described above, except challenging of DCs with HLA-A0201-restricted Wilms’ tumor-derived peptide WT1-A (10 µg/ml; 126–134; PRIMM, Italy) for 4 h. DCs loaded with WT1-A or WT1-B (187–195) were used as targets.

### *In Vitro* Tregs Induction

Immature DCs, chemotherapy-treated HL-60 pulsed DCs and DCs matured in presence of ATP or a cytokine cocktail were co-cultured for 5 days in autologous RPMI with allogeneic CD3^+^ T cells, in presence or absence of the IDO1-inhibitor 1-methyl tryptophan-L (1 mM, 1-MT-l; Sigma-Aldrich). IDO1-silenced DCs were also used (see Datasheet S1 Supplementary Material) (10). Tregs were quantified by FACS analysis. In selected experiments, purified and irradiated CD4^+^CD25^+^CD127^dim/−^ Tregs (10^4^/well) were added to cultures consisting of CFSE-labeled CD3^+^ T cells (10^5^/well) as responders, stimulated by allogeneic immature monocyte-derived DCs (1:10 ratio), for 5 days, to test their suppressive activity.

### Flow Cytometry on Human Cells

T cells, Tregs, and DCs were characterized using the following mAbs, according to manufacturer’s instructions: anti-CD8 Pacific blue (B9.11) or PE, anti-CD15S PE-CF594 (CSLEX1), anti-CD25 BV605 (2A3), anti-CD28 APC or APC-eFluor 780 (CD28.2), anti-CD80 PE-Cy 7 (L307.4), anti-CD152 (CTLA-4; clone BNI3), and anti-Ki-67 Alexa Fluor 700 (B56) from BD Bioscience; anti-CD3 PE-Cy7 or APC (UCHT1) or APC-eFluor 780 (SK7), anti-CD4 FITC (RPA-T4), anti-CD8 APC (SK1), anti-CD25 APC (SK1) or APC-eFluor 780 (CD25-4E3), anti-CD38 Alexa 700 (HIT2), anti-CD83 PE (HB15e), anti-CD127 Pe-Cyanine5 (eBioRDR5), anti-CD197 (CCR7) PE-Cy7 or Brillant Violet 421 (G043H7), and anti-HLA-DR FITC (L243) from eBioscience; anti-CD4 FITC (SFCI12T4D11) and anti-CD45RA ECD (2H4LDH11LDB9) (Beckman Coulter, USA); anti-CD39 PE/Cy7 (A1), anti-CD45RA Brilliant Violet 510 (HI100), anti-CD127 PerCP/Cy5.5 (IL-7Rα; clone A019D5), and anti-CD279 (programmed cell death protein 1; PD-1) APC or Brilliant Violet 711 (EH12.2H7) from Biolegend. LIVE/DEAD Fixable Aqua (Thermo Fisher Scientific, USA) was used to gate out dead cells. For intracellular staining (24), after cells fixation and permeabilization with 4% paraformaldehyde (VWR, USA) and 0.1% saponin (Sigma), the following mAbs were used: anti-IFN-γ PE (4S.B3), anti-Foxp3 PE or FITC (236A/E7), and anti-Ki-67 FITC (20Raj1) from eBioscience. Circulating Tregs in PB of AML patients were assessed by using Human Regulatory T Cell Whole Blood Staining Kit (eBioscience) (see Datasheet S1 in Supplementary material).

Specifically, for *ex vivo* post-chemotherapy patients’ Tregs screening, anti-CD3/CD4/CD25/FOXP3 mAbs were used. In selected cases (*n* = 6), a wider panel including anti-CD3/CD4/CD25/CD127/CD15s/CD45RA/FOXP3/PD-1/Ki67 mAbs for identification of Tregs subpopulation (Treg1/2/3) were used.

For each sample, isotype-matched irrelevant mAbs staining was used as control. At least 10,000 events were collected from each sample at Gallios Flow Cytometer (Beckman Coulter) or BD Accuri C6 (BD Biosciences). For expanded T cells, data analyses were performed by using FlowJo Single Cell Analysis Software (FlowJo LLC).

### *In Vivo* Studies

To obtain WEHI-3B cell clones stably expressing plasma membrane luciferase, cells were transfected with the PmeLUC probe, as previously described (25). Briefly, 6 × 10^6^ cells were suspended in electroporation buffer (Life Technologies, USA) with 3 µg of plasmid’s DNA and electroporated in a Microporator MP-100 (Digital bio, Thermo Fisher), at 1,250 V for 40 ms. Stably transfected cell clones were obtained by selection with neomycin/G418 sulfate (0.2–0.8 mg/ml; Sigma) followed by limiting dilution as previously described (26). Male 4–6 weeks old Balbc/J wt or P2X7^−/−^ mice were subcutaneously injected with 2 × 10^6^ WEHI-3B PmeLUC cells. Tumors became palpable approximately 7 days post-inoculum (p.i.) and were measured with a manual caliper. Tumor volume was calculated according to the following equation: π/6 [*w*1 × (*w*2)^2^], where *w*1 is the major diameter and *w*2 is the minor diameter. Luminescence emission was measured daily, days 7–12 from p.i. with a total body luminometer for small animals (IVIS Lumina, Caliper; Perkin Elmer, USA), as previously described (27). Mice anesthetized with 2.5% isofluorane were intra peritoneum (i.p.) injected with 150 mg/kg d-luciferin (Promega, USA) and luminescence was captured from dorsal view. Photon emission was quantified using the Living Image^®^ software (Perkin Elmer) and averaged as photons/second/cm^2^/steradian (abbreviated as p/s/cm^2^/s). DNR (3 mg/kg, Sigma), ARA-C (50 mg/kg, Sigma), or sterile PBS vehicle (placebo) were i.p. administered at p.i. days 7 and 9. Blood samples were collected from the submandibular vein under general anesthesia immediately before sacrificing the animal (p.i. day 12). Cytokines levels were evaluated following 1:2 plasma dilution with Ciraplex CK1 mouse multi-cytokine assay kit (AushonBiosystem, distributed by TemaRicerca, Bologna, Italy) as per manufacturer’s instructions. Tumors were excised and processed for flow cytometry (see Datasheet S1 in Supplementary material). All animal procedures were approved by the University of Ferrara (Ferrara, Italy) Ethic committee and the Italian Ministry of Health in compliance with International laws and policies (EU Directive 2010/63/EU and Italian D.Lgs 26/2014; authorization number 821/2015PR to EA).

### Statistical Analysis

Data were expressed as mean ± SEM of values obtained in the experiments. Statistical analyses were performed with GraphPad Prism 6 software (GraphPad Software, Inc., La Jolla, CA, USA), using ANOVA or unpaired *t*-test. *p* Values <0.05 were considered statistically significant.

## Results

### Increased Tregs with Suppressive Phenotype Are Detectable in AML Patients after Combined DNR and ARA-C Chemotherapy

We analyzed the induction of tumor-reactive CD8^+^ cytotoxic T cells (CTLs) in a cohort of AML patients (*n* = 23) undergoing combined DNR and ARA-C chemotherapy. In 15 out of 23 patients, we observed an increase of leukemia-reactive IFN-γ-producing CD8^+^ T cells (Figure S1 in Supplementary Material) mostly belonging to effector memory (EM) and EM expressing CD45RA (effector memory expressing RA) subsets (Figure S2 in Supplementary Material), which highly expressed the activation marker CD38 and downregulated CD28 as compared to naïve (*p* = 0.03) or central memory (CM) (*p* = 0.03) T cells.

**Figure 1:**
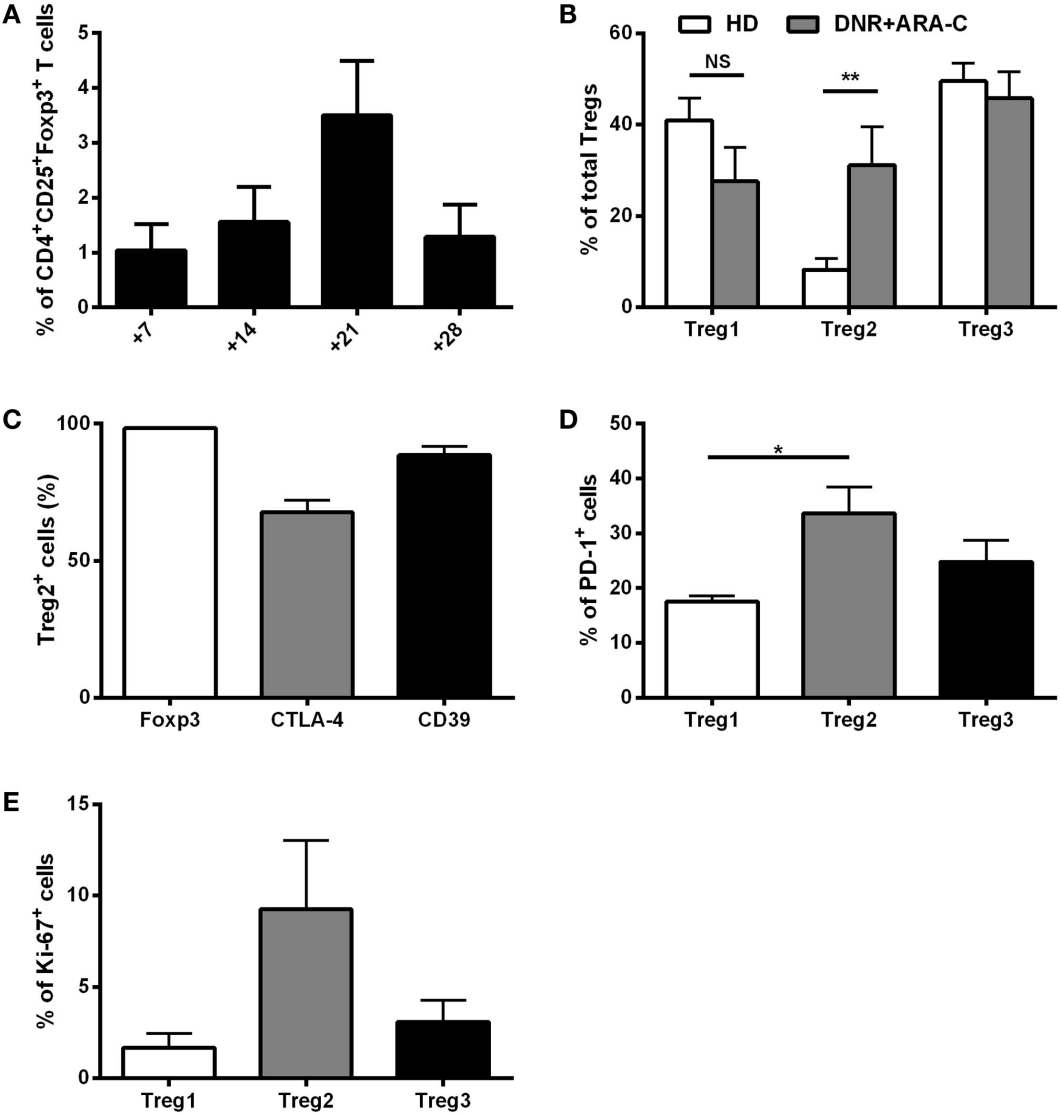
*Ex vivo* characterization of T regulatory cells (Tregs) after DNR + ARA-C chemotherapy. **(A)** Percentage of circulating CD4^+^CD25^+^Foxp3^+^ Tregs in peripheral blood of 23 acute myeloid leukemia (AML) patients at days 7, 14, 21, and 28 after chemotherapy analyzed by flow cytometry. **(B)** Characterization of Tregs subsets based on the expression of CD45RA and CD15s cell surface markers in AML patients at day 21 by flow cytometry (DNR + ARA-C). Total Tregs (gated on CD3^+^CD4^+^CD25^+^CD127^dim/−^ T cells) are subdivided into Treg1 (CD45RA^+^CD25^+^CD15s^−^), Treg2 (CD45RA^−^CD25^+^CD15s^+^), and Treg3 (CD45RA^−^CD25^+^CD15s^−^) (*n* = 6). Healthy donors (HD) were used as negative control (*n* = 6). **(C)** Expression of Foxp3, CTLA-4, and CD39 markers in Treg2 subset of AML patients at day 21 by FACS analysis (*n* = 6). Expression of PD-1 **(D)** and Ki-67 **(E)** markers in the different subsets of Tregs by FACS analysis (*n* = 6). *p < 0.05; **p < 0.01; NS, not significant difference. Values are represented as mean ± SEM.

Along with the detection of leukemia-reactive CD8^+^ T cells, we observed an increase in CD4^+^CD25^+^Foxp3^+^ T cells after DNR plus ARA-C chemotherapy (Figure 1A) with a peak at day 21. We, then, sought to better characterize Tregs subsets at the phenotypic level. Among CD3^+^CD4^+^ T cells collected at day 21 post-chemotherapy, CD25^+^CD127^−^ cells were subdivided into three different subsets (Treg1, Treg2, Treg3), according to the expression of CD15s (Figure S3 in Supplementary Material) (28, 29). As compared to HD, no differences were observed for Treg1 and Treg3 frequencies, whereas the percentage of Treg2 cells was significantly increased in AML patients after chemotherapy (Figure 1B). Treg2 cells from AML patients expressed Foxp3, which correlated with CTLA-4 and CD39 (Figure 1C), indicating a suppressive phenotype (28, 30, 31). Moreover, as compared to Treg1, Treg2 cells showed higher expression of PD-1, which identifies a novel population of Tregs with crucial suppressive activity in the tumor setting (Figure 1D) (32, 33). Intriguingly, after 21 days post-chemotherapy an increase in Ki67 expression was observed in Treg2 over Treg1 and Treg3 cells, suggesting recent activation (Figure 1E). Indeed, a time-course analysis revealed a selective increase of proliferating Ki67^+^ Treg2 over Treg1 and Tregs3 cell subsets at day 14 post-chemotherapy, which progressively reduced at later time points (Figure S4 in Supplementary Material).

Taken together, the analysis of the composition of T cells, emerging in AML patients after combined DNR and ARA-C chemotherapy, reveals an increase of Tregs with a suppressive phenotype. These *ex vivo* data prompted us to dissect the contribution of DNR versus ARA-C in Tregs induction and to investigate a possible mechanism responsible for chemotherapy-driven induction of a suppressive microenvironment in AML.

### *In Vitro*, DNR Is More Effective Than ARA-C at Inducing Fully Functional Tregs through DCs

**Figure 2:**
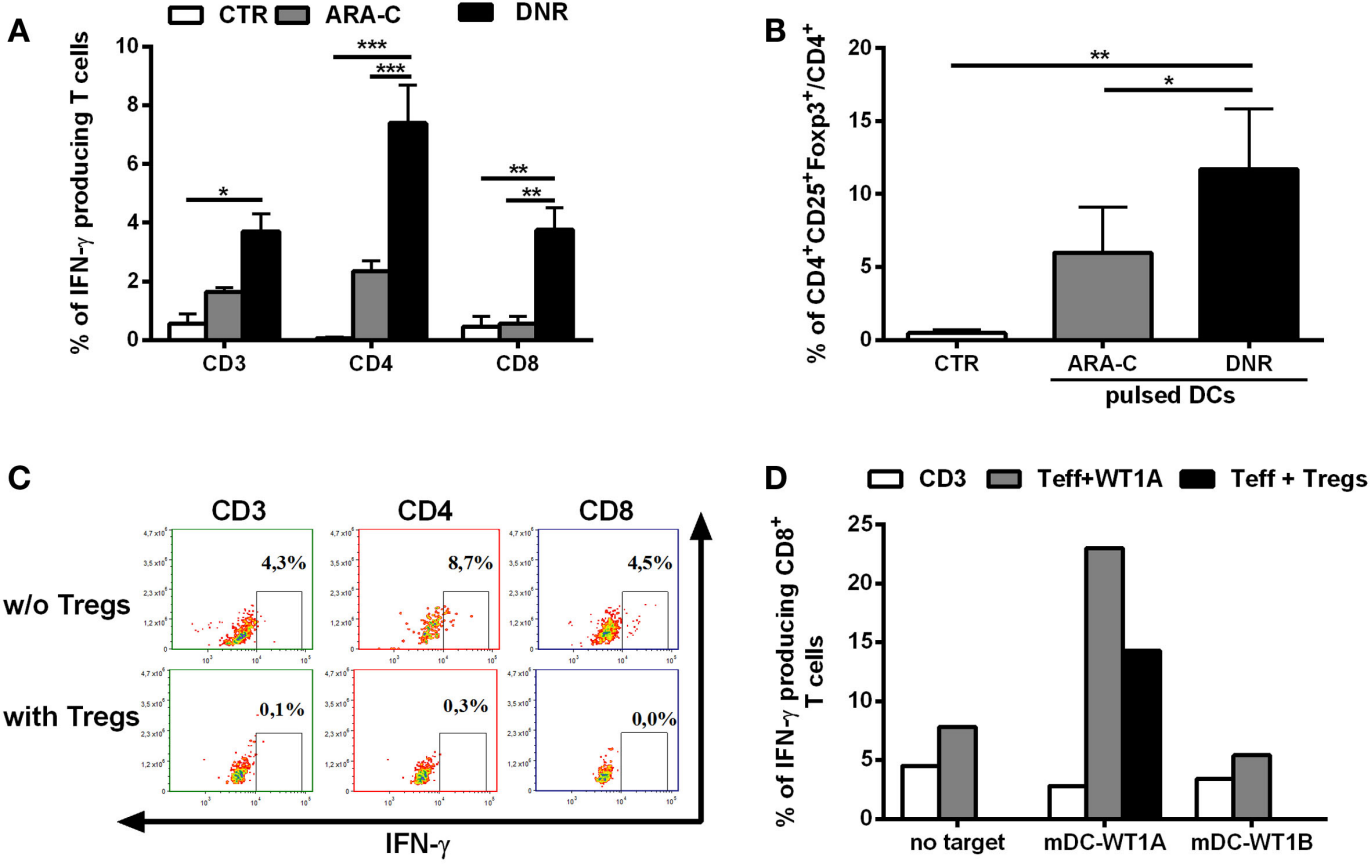
DNR is more efficient than ARA-C in promoting induction of functional T regulatory cells (Tregs). **(A)** Percentage of IFN-γ-producing CD3^+^, CD4^+^, and CD8^+^ leukemia-reactive T cells, previously stimulated and restimulated with DCs loaded with ARA-C- or DNR-treated HL-60 cells (ARA-C or DNR, respectively), evaluated by flow cytometry. Autologous DCs loaded with HL-60 cell lysate were used as target. Unstimulated CD3^+^ T cells were used as negative control of T effector cells (CTR). The values are represented as mean ± SEM of three independent experiments; *p < 0.05; **p < 0.01; ***p < 0.001. **(B)** Percentage of CD4^+^CD25^+^Foxp3^+^ T cells (gated on CD4^+^ T cells) induced by DCs pulsed with ARA-C- or DNR-treated HL-60 cells. Unstimulated CD3^+^ T cells were used as negative control (CTR); *p < 0.05; **p < 0.01. Values are represented as mean ± SEM of three independent experiments. **(C)** FACS analysis of inhibition of CD3^+^, CD4^+^, and CD8^+^ IFN-γ-producing T cells by autologous CD4^+^CD25^+^Foxp3^+^ Tregs. One representative experiment of three is shown. **(D)** Quantification of IFN-γ-producing leukemia antigen-specific CD8^+^ T cells, previously stimulated and restimulated with mature DCs loaded with WT1-A (Teff WT1A), in presence (Teff + Tregs) or absence of autologous CD4^+^CD25^+^Foxp3^+^ Tregs. Autologous DCs loaded with WT1-A or WT1-B (as antigen specificity control) were used as targets (mDC-WT1A or mDC-WT1B, respectively), T cells without target (no target) were used as negative control. Unstimulated CD3^+^ T cells were used as negative control of effector cells (CD3). Tregs were added to T effector cells at ratio of 1:1. One representative experiment of three is shown.

Inflammatory stimuli may promote Tregs *via* the induction of tolerogenic DCs (16). Since chemotherapy treatment is associated with the abundant release of inflammatory signals, we tested the contribution of DNR and ARA-C in driving DCs toward Tregs induction. Chemotherapy-treated AML cells were pulsed into DCs, which were, then, used in co-culture for inducing leukemia-specific T cells and Tregs. DC-loaded with DNR-treated AML cells were more efficient than DC-loaded with ARA-C-treated cells not only, as expected, in inducing leukemia-reactive CD8^+^ T cells (Figure 2A), but also in increasing the frequency of CD4^+^CD25^+^Foxp3^+^ T cells (11.70 ± 4.15 versus 5.97 ± 3.10%, respectively; *p* < 0.05) (Figure 2B). DNR-induced CD4^+^CD25^+^Foxp3^+^ T cells were shown to act as Tregs through the complete inhibition of leukemia-specific IFN-γ production by both CD4^+^ and CD8^+^ T cells (Figure 2C). Moreover, these DC-induced Tregs also reduced T cell-mediated IFN-γ-production in response to the leukemia-associated antigen, WT1 (Figure 2D).

These results demonstrate that DNR is more efficient than ARA-C in promoting functional Tregs through tolerogenic DCs.

### *In Vitro*, ATP Release from DNR-Treated Leukemia Cells Is Correlated with Tregs Generation and with IDO1 Upregulation in Mature DCs

**Figure 3:**
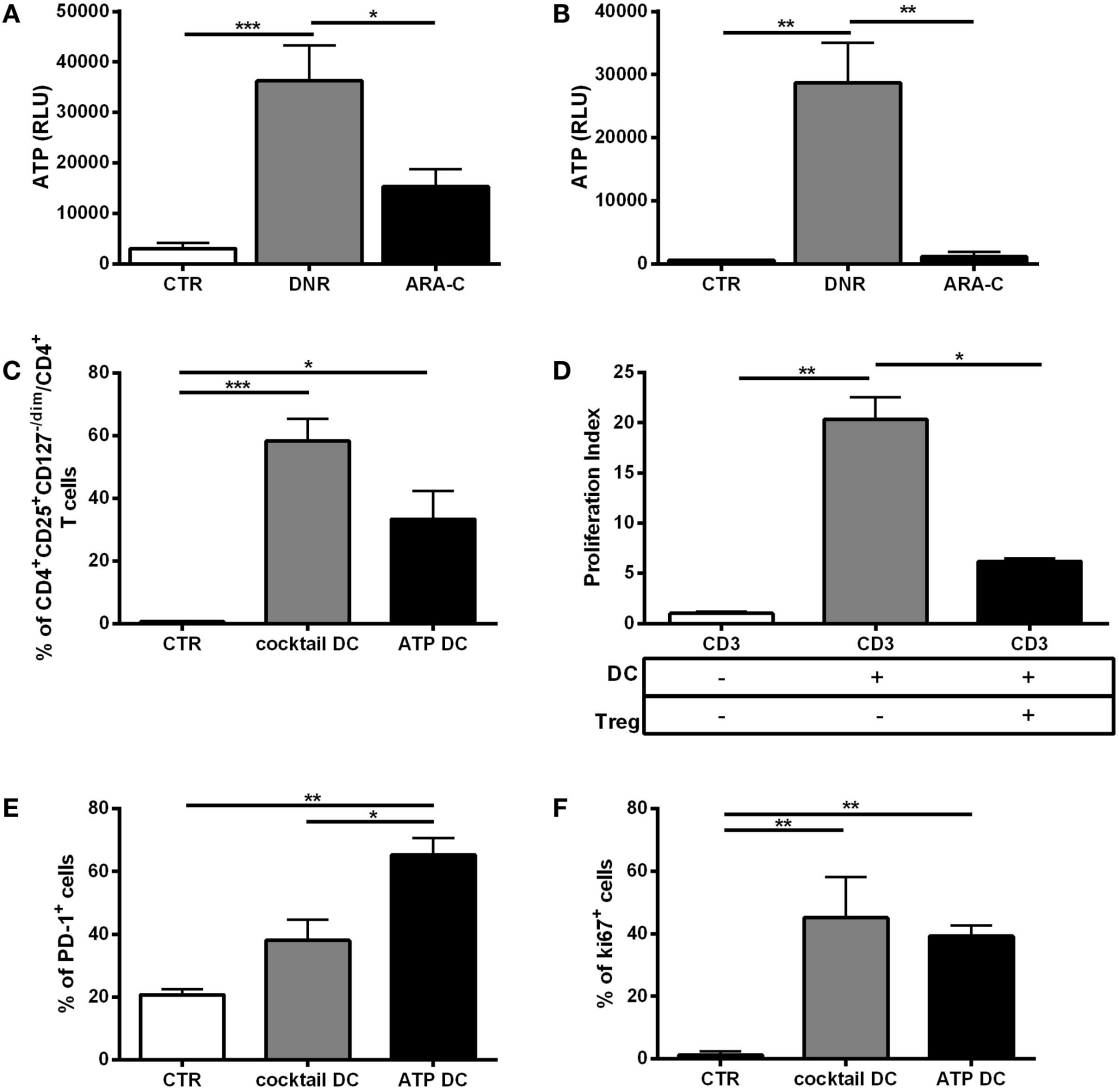
*In vitro*, ATP release from DNR-treated leukemia cells is correlated with functional T regulatory cells (Tregs) generation. Indirect measurement of ATP by quantification of emitted bioluminescence, expressed as relative light unit (RLU) in supernatants of **(A)** HL-60 and **(B)** primary acute myeloid leukemia cells after DNR (500 ng/ml) and ARA-C (25 µg/ml) treatment or untreated (CTR). The values are represented as mean ± SEM of four independent experiments; *p < 0.05; **p < 0.01; ***p < 0.001. **(C)** Percentage of Tregs identified as CD4^+^CD25^+^CD127^−/dim^ cells induced after 5 days of co-culture of ATP- or cytokine cocktail-treated dendritic cells (DCs) (ATP DC or cocktail DC, respectively) with allogeneic CD3^+^ T cells. Unstimulated CD3^+^ T cells were used as negative control (CTR). The values are represented as mean ± SEM of three independent experiments; *p < 0.05; ***p < 0.001. **(D)** To test their immunosuppressive activity, CD4^+^CD25^+^CD127^dim/−^ Tregs obtained at the end of the co-culture with ATP DC (Treg) were purified and added (1:10) to CD3^+^ T cells stimulated with mature DCs (DC). Proliferation index was evaluated by FACS analysis of CFSE staining. Unstimulated CD3^+^ T cells were used as negative control. Histograms represent the means ± SEM of three independent experiments; *p < 0.05; **p < 0.01. **(E)** Expression of PD-1 cell surface marker and **(F)** intracellular Ki-67 proliferation marker in CD4^+^CD25^+^CD127^−/dim^ Tregs induced by ATP-treated DCs. DCs matured with cytokine cocktail were used as positive control, whereas CD3 alone was used as negative control (*n* = 3); *p < 0.05; **p < 0.01.

Within tumor microenvironment, chemotherapy-treated tumor cells release a high amount of ATP, which is a major driver of DC activation and function (5, 6). To test the contribution of ATP release from chemotherapy-treated AML cells in DC-mediated Tregs induction, we preliminarily *in vitro* measured ATP release from dying AML cells, including cell lines and primary cells, after treatment with DNR and ARA-C. DNR and ARA-C were used at the concentrations capable to induce comparable apoptosis level, but only DNR increased ATP release (Figures 3A,B). These data suggest that the increased capacity of DNR over ARA-C to induce Tregs *via* DCs may be, at least in part, mediated by ATP. Indeed, when treated with ATP, DCs induced a population of CD4^+^CD25^+^CD127^−/dim^ T cells (Figure 3C), which acted as *bone fide* Tregs by reducing allogeneic T-cell proliferation (Figure 3D). Interestingly, in comparison to a cocktail of pro-inflammatory cytokines, ATP treatment of DCs resulted in higher expression of PD-1 on Tregs (Figure 3E), whereas Foxp3 and CD39 expression was similar (data not shown). Moreover, Tregs obtained after culture with ATP-treated DCs expressed Ki-67 at comparable level, suggesting recent activation/proliferation (Figure 3F).

**Figure 4:**
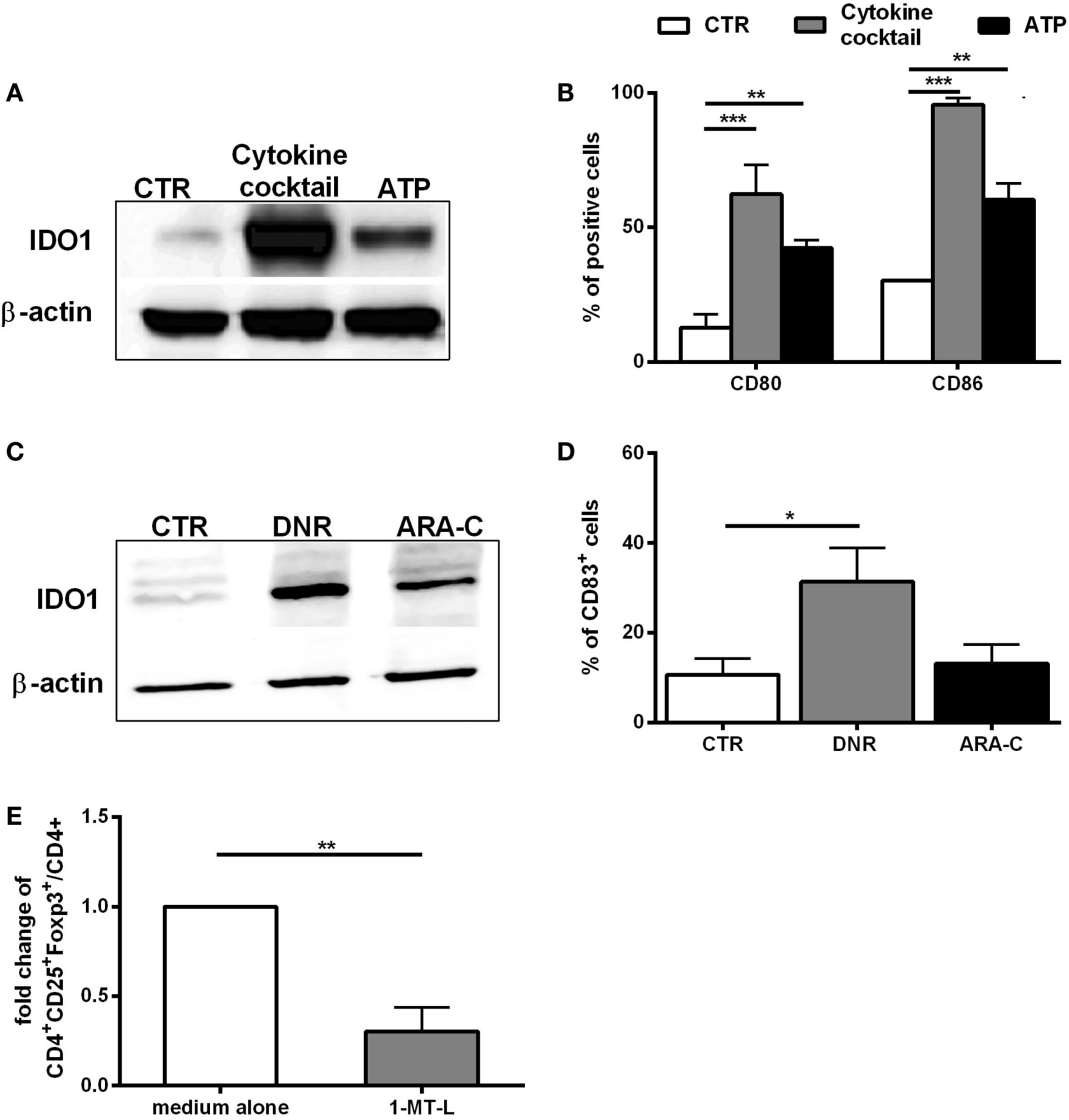
*In vitro*, ATP release from DNR-treated leukemia cells is correlated with IDO1 upregulation in mature dendritic cells (DCs). **(A)** Western blot analysis of IDO1 protein expression in DCs treated with ATP (1 mM; 24 h). Unloaded DCs and DCs matured with cytokine cocktail were used as negative (CTR) and positive control (cytokine cocktail). One representative experiment is shown. **(B)** FACS analysis of CD80 and CD86 expression in DCs treated as described in panel **(A)**. The percentage values are represented as mean ± SEM of three independent experiments; **p < 0.01; ***p < 0.001. **(C)** Western blot analysis of IDO1 protein expression in DCs loaded with DNR- or ARA-C-treated HL-60 cells (DNR 500 ng/ml, ARA-C 25 µg/ml). Unloaded DCs were used as negative control (CTR). One representative experiment is shown. **(D)** Expression of CD83 maturation marker in DCs treated as described in panel **(C)**. The percentage values are represented as mean ± SEM of four independent experiments; *p < 0.05. **(E)** Fold change of CD4^+^CD25^+^Foxp3^+^ T cells (gated on CD4^+^ T cells) induced by DCs loaded with DNR-treated HL-60 cells in presence of 1-MT-l. CD4^+^CD25^+^Foxp3^+^ T cells induced by DCs in absence of 1-MT-l (medium alone) were used as reference and set as 1; **p < 0.01.

Among the different mechanisms used by DCs for promoting Tregs, IDO1 upregulation is crucial (15, 16). Then, we investigated the involvement of ATP in IDO1 upregulation in DCs during chemotherapy. ATP treatment upregulated IDO1 protein along with the maturation markers, CD80 and CD86 (Figures 4A,B), albeit to a lower extent than pro-inflammatory cytokines, used as positive control (21). Of note, in agreement with the different levels of ATP release (Figures 3A,B), DNR was more efficient than ARA-C in upregulating IDO1 protein (Figure 4C), along with the maturation marker CD83, which was significantly increased by DNR, and not by ARA-C treatment (*p* < 0.05) (Figure 4D). The inhibition of IDO1 in DCs upon DNR treatment by the IDO1-inhibitor, 1-MT-l (Figure 4E, *p* < 0.01) or by IDO1-specific RNA interference (data not shown) significantly reduced DC-mediated Tregs induction, thus demonstrating that DNR-induced IDO1 upregulation in DCs may drive Tregs. Taken together, these data suggest that ATP release from DNR- and not ARA-C-treated leukemia cells is correlated with Tregs generation and IDO1 upregulation in mature DCs.

### DNR-Treated Mice Show Increased Level of ATP Release from Leukemia Cells

We, then, investigated in a mouse model the link between ATP release from chemotherapy-treated cells and the induction of an immune suppressive microenvironment. In line with data obtained with human AML cells (Figures 3A,B), only DNR, and not ARA-C, increased the release of ATP from murine WEHI-3B AML cells *in vitro* (Figure S5 in Supplementary Material).

**Figure 5:**
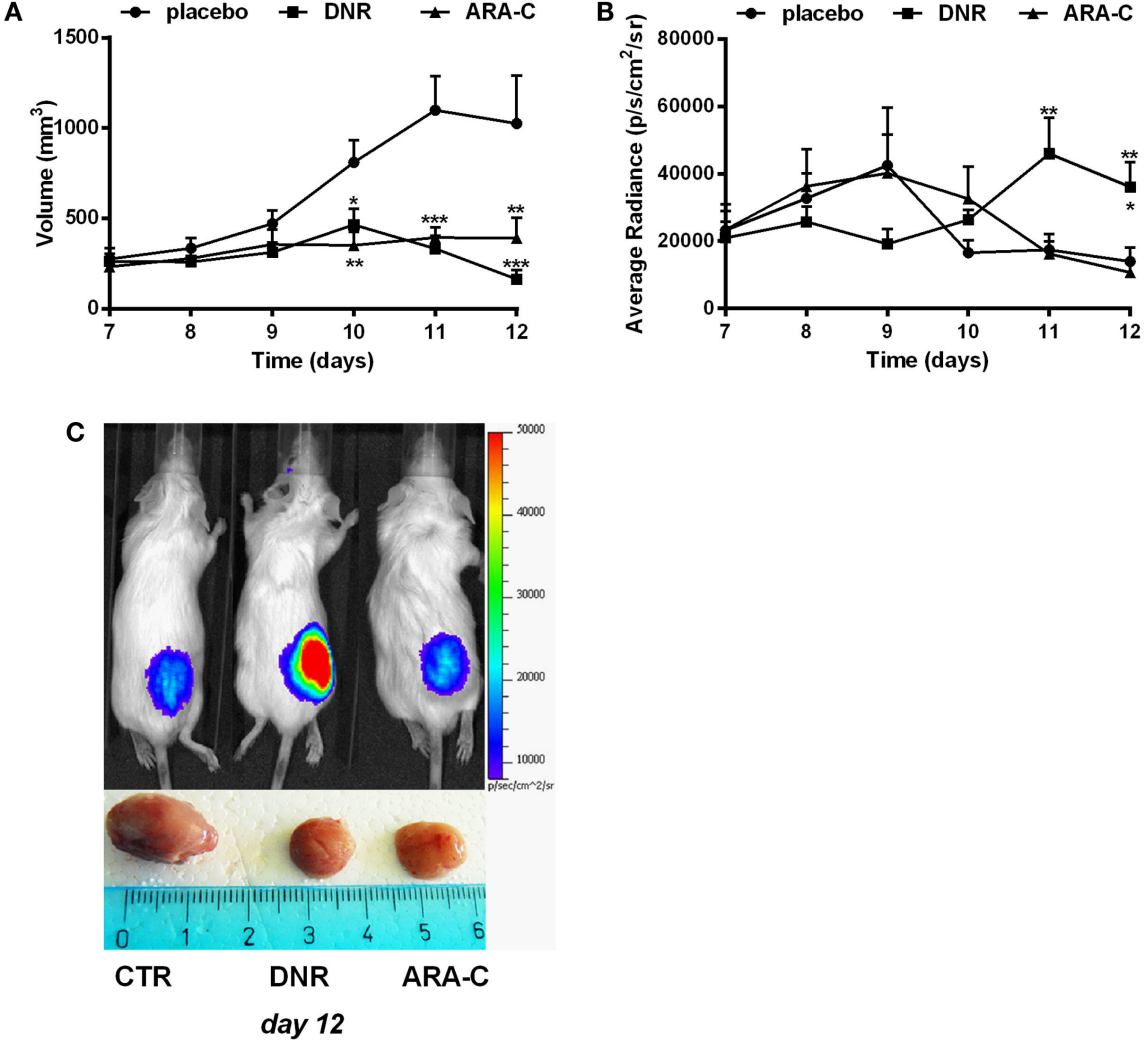
DNR increases ATP release *in vivo*. Balb/cJ mice inoculated with WEHI-3B PmeLUC cells and treated with DNR, ARA-C, or PBS vehicle (placebo) at post-inoculum days 7 and 9. **(A)** Tumor volume was *in vivo* assessed by a caliper and calculated as follows: π/6 [*w*1 × (*w*2)^2^] (*w*1 = major diameter, *w*2 = minor diameter). **(B)** Kinetic measurements of ATP levels in treated Balb/cJ mice estimated by PmeLUC luminescence. Reported data represent the average number of photons in the tumor area (p/s/cm^2^/s = photons/second/cm^2^/steradian). The values are represented as mean ± SEM of 12 mice per condition; *p < 0.05; **p < 0.01; ***p < 0.001 versus placebo and, when reported, versus ARA-C. **(C)** Representative pictures (*n* = 3) of PmeLUC luminescence emission in treated Balb/cJ mice and of *ex vivo* tumor volumes at post-inoculum day 12.

This cell line was then transfected with PmeLUC, a luciferase expressed at the plasma membrane, which allows for ATP measure in the extracellular milieu (25). WEHI-3B PmeLUC live clones were injected in syngeneic mice, which were then treated with either DNR or ARA-C. While DNR and ARA-C treatment had comparable effect on tumor mass reduction (Figure 5A), only DNR increased ATP release (36,094.20 ± 7,420.75 p/s/cm^2^/sr) over control (13,950.9 ± 4,118.65 p/s/cm^2^/sr; *p* < 0.05) (Figures 5B,C). These data prompted us to correlate the composition of leukemia immune suppressive microenvironment, i.e., Tregs and tolerogenic DCs, with the different levels of ATP released upon chemotherapy.

### ATP Release from DNR-Treated Mice Increases Leukemia-Infiltrating Tregs and IDO1-Expressing DCs

**Figure 6:**
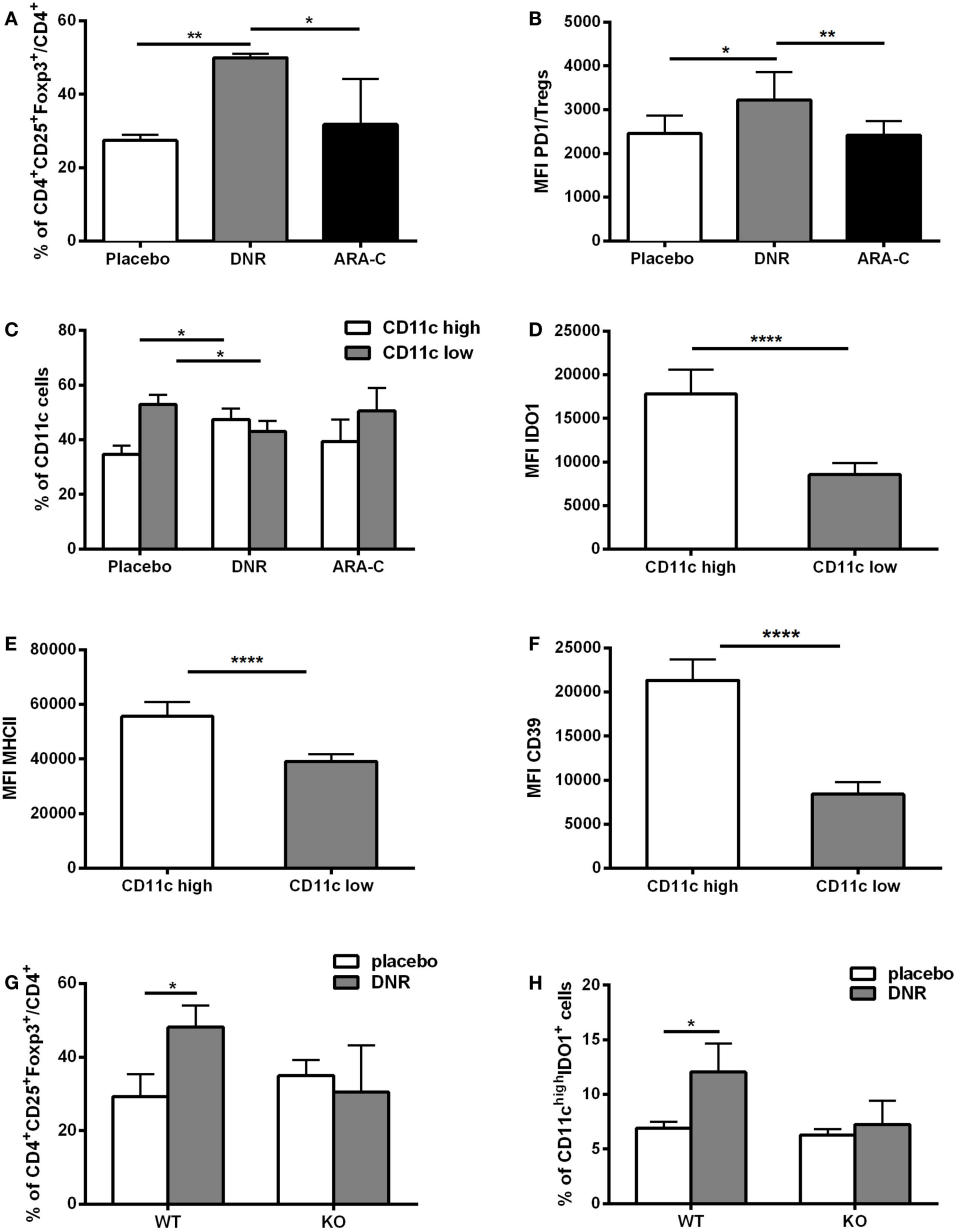
ATP release from DNR-treated mice increases leukemia-infiltrating T regulatory cells (Tregs) and IDO1-expressing dendritic cells (DCs). **(A)** Percentage of CD4^+^CD25^+^Foxp3^+^ infiltrating T cells (gated on CD4^+^ T cells) by FACS analyses. Data were obtained from six mice per condition. Mice were treated with placebo, DNR, or ARA-C as described in Figure 5; *p < 0.05; **p < 0.01. **(B)** The mean fluorescence intensity (MFI) of PD-1 in CD4^+^Foxp3^+^ infiltrating T cells (Tregs); *p < 0.05; **p < 0.01. **(C)** Percentage of infiltrating CD11c^low^ and CD11c^high^ myeloid DCs by FACS analyses. Data were obtained from six mice per condition; *p < 0.05. MFI obtained by flow cytometry indicates the expression level of IDO1 **(D)**, MHCII **(E)**, and CD39 **(F)** in CD11c^low^ and CD11c^high^ myeloid DCs in DNR-treated mice; ****p < 0.0001. FACS analysis of tumor-infiltrating CD4^+^CD25^+^Foxp3^+^ T cells **(G)** and CD11c^high^IDO1^+^ myeloid DCs **(H)** in P2X7 wild-type (WT) and knockout (KO) DNR- or placebo-treated mice. Data were obtained from six mice per condition and represented as mean ± SEM.

Chemotherapy-induced ATP release correlates with an increase in pro-inflammatory mediators (34, 35). Accordingly, administration of DNR but not ARA-C promoted a significant increase of IFN-γ (*p* < 0.05), IL-1β (*p* < 0.01), IL-2 (*p* < 0.001), and IL-12 (*p* < 0.05) in the serum of leukemia-bearing mice (Figure S6 in Supplementary Material). To investigate the capacity of chemotherapy to elicit a suppressive, along with its activatory, effect, leukemia infiltrate was analyzed after DNR and ARA-C treatment and compared to placebo. Among CD4^+^ T cells, DNR treatment was associated with a significant increase of total CD25^+^Foxp3^+^ T cells as compared to placebo and ARA-C (Figure 6A). Notably, DNR, and not ARA-C, significantly increased the expression of PD-1 on CD4^+^CD25^+^Foxp3^+^ T cells, both as percentage of positive cells (data not shown) and as mean fluorescence intensity (Figure 6B). This finding in DNR-treated mice paralleled with the increase of PD-1-expressing Tregs in AML patients after induction chemotherapy (Figure 1D) and in Tregs obtained *in vitro* after culture with human ATP-treated DCs (Figure 3E). Moreover, among CD11b^+^MHCII^+^LY6C^−^ myelo-monocytic cells (Figure S7 in Supplementary Material) only DNR, and not ARA-C treatment upregulated DC expression of CD11c (Figure 6C), which correlated with higher expression of IDO1 (Figure 6D) and MHCII (Figure 6E). Interestingly, in DNR-treated mice, CD11c^high^ DCs had also increased expression of CD39 (Figure 6F), which is the rate-limiting enzyme during ATP catabolism (36, 37).

To demonstrate *in vivo* the contribution of ATP release in increasing the infiltration by Tregs and tolerogenic DCs after DNR treatment, P2X7R null mice were injected with WEHI-3B PmeLUC cells and treated with DNR. The levels of ATP after DNR treatment were similar to those observed in wild-type (WT) mice (Figure S8 in Supplementary Material). However, differently from WT mice, in P2X7 null mice DNR failed to increase CD4^+^CD25^+^Foxp3^+^ Tregs (Figure 6G) and IDO1-expressing CD11c^high^ DCs (Figure 6H). Taken together, these *in vivo* results demonstrate that ATP release from DNR-treated dying leukemia cells has a role in the induction of an immune suppressive microenvironment, which comprises Tregs and IDO1-expressing DCs.

## Discussion

Some chemotherapeutic agents, such as DNR, induce immunogenic cell death of both solid tumors (1–3, 38) and leukemias (1, 39), allowing immune recognition and T-cell-mediated anticancer response. However, this treatment is rarely curative, especially in AML, suggesting that other mechanisms are in place. One possibility is that, besides inducing T-cell activation, such treatment could concomitantly promote the development of immune suppressive pathways, not yet exploited (7, 8). Accordingly, our data from AML patients indicate that CD8^+^ T cells emerging after combined DNR and ARA-C chemotherapy are not only functionally capable of leukemia recognition but are also coupled with a novel population of Tregs with suppressive phenotype, Treg2 cells, recently described according to CD15s (28, 29). Tregs are known to induce a suppressive state in tumor-infiltrating CTLs, which favors leukemia development and growth (40–42). Clinically, in AML, the persistence of increased Tregs after chemotherapy is correlated with poor clinical outcome (43), which is paralleled with the exhaustion of leukemia-specific CTLs over time in association with disease relapse (44, 45).

It has been reported that ATP regulates the function of lymphoid cells with opposite effects depending on the cellular subsets analyzed (11, 46, 47). In case of Tregs, ATP may directly elicit their suppressive function or may be metabolized by ecto-5′-nucleotidases, such as CD39 and CD73, into adenosine, which enhances Tregs activity (11–13). Our results indicate that, during chemotherapy, the levels of ATP within leukemia microenvironment are positively correlated with the frequency of Tregs. Indeed, only DNR treatment, which was capable of significantly augmenting intra-leukemia ATP level, was associated with increased Tregs. In mice lacking the ATP receptor P2X7R, DNR failed to increase Tregs, strongly supporting the crucial role of ATP in this process. Notably, *in vivo* ATP-stimulated Tregs showed higher expression of the immune checkpoint inhibitor PD-1, similarly to PD-1-expressing suppressive Treg2 cells expanded in chemotherapy-treated AML patients. Upon inflammation, the triggering of PD-1 on T cells negatively regulates their proliferation and cytokine production (48–50). The expression of PD-1 on Tregs correlates with their immunosuppressive activity (32, 33, 51) and the accumulation of PD1^+^Foxp3^+^ Tregs within the tumor microenvironment of solid tumors further confirms their immunosuppressive potential (32, 51). Our data confirm and extend these data to AML and provide new evidence and explanation for the correlation between the frequency of PD1^+^ Tregs and the level of ATP released from chemotherapy-treated leukemia cells.

ATP release from chemotherapy-treated tumor cells is a potent driver of antitumor immune response through P2X7R activation on DCs, which are induced to complete maturation and full competence in antigen presentation (6, 52, 53). Whether chemotherapy-dependent ATP release may also mediate the induction of tolerogenic features in tumor-infiltrating DCs has been poorly investigated. Our data add evidence to the hypothesis that ATP release from chemotherapy-treated tumor cells, being a potent inflammatory stimulus, may have a dual role on DC function. Along with the well-established effect on DC function toward cross-priming (53, 54), ATP may also promote the induction of tolerogenic pathways, such as IDO1. Our previous work in AML demonstrated that IDO1 mediates the conversion of naive CD4^+^CD25^−^ T cells into fully competent Tregs, and that leukemic DCs inhibit the induction of leukemia-specific T cells *via* IDO1 (10, 19). Here, we show that ATP release from chemotherapy-treated AML cells drives IDO1 upregulation in DCs in a P2X7R-dependent manner. These DCs are *in vitro* fully competent at inducing through IDO1 a population of Tregs, which in turn inhibit leukemia-specific T-cell immune response. According to a previous report (51), upon chemotherapy the effect of ATP on IDO1 expression is part of a continuum, in which the DC maturation process may stand by IDO1 production to counterbalance the exceeding immune activation triggered by pro-inflammatory stimuli and associated to chemotherapy-induced immunogenic cell death (16, 18). Of note, our *in vivo* experiments indicate that during chemotherapy increased levels of ATP are associated with higher expression of CD39 on DCs, which regulates the rate-limiting enzymatic step of ATP catabolism (37). Such correlation is intriguing and may suggest that, within an ATP-enriched tumor microenvironment, infiltrating DCs may take part to ATP degradation by modulating on their surface the expression of ATP-specific ecto-nucleotidases.

Overall, our data indicate that chemotherapy treatment for AML, along with its well-known immunogenic effect, may also result in the induction of an immune suppressive microenvironment. This scenario, at least in part, depends on the local effects of ATP release from dying leukemia cells, which may critically influence an increase of leukemia-associated Tregs and tolerogenic DCs. A better understanding of the mechanisms leading to the induction of a leukemia immune suppressive microenvironment during chemotherapy has important clinical implications to fully exploit the immunogenic potential of anti-leukemia agents and tune their application.

## Ethics Statement

### For Human Samples

This study was carried out in accordance with the recommendations of Ethics Committee of the University Hospital of Bologna S. Orsola-MALPIGHI with written informed consent from all subjects. All subjects gave written informed consent in accordance with the Declaration of Helsinki. The protocol was approved by the Ethics Committee of the University Hospital of Bologna S. Orsola-Malpighi, approval code: 147/2013/O/Tess.

### For Animal Subjects

This study was carried out in accordance with the recommendations of Ethical Committee of University of Ferrara. The protocol was approved by the Ethical Committee of University of Ferrara and Italian Ministry of Health (EU Directive 2010/63/EU and Italian D.Lgs 26/2014; authorization number 821/2015PR to EA).

## Author Contributions

ML and DO performed *ex vivo* characterization of lymphocytes, cell cultures experiments, and *in vitro* assays, analyzed the data, and wrote the manuscript; VS and AR analyzed IFN-γ-producing lymphocytes from patients; EM, EO, SS, AP, PP, and AB performed *in vivo* assays and analyzed the data; ST and CJ performed *ex vivo* characterization of lymphocytes and cytotoxicity test; AB, PR, and MC contributed to write the paper; MPC and FV contributed to study design and to write the paper; EA and AC designed the research, analyzed the data, wrote the manuscript, and gave the final approval for submission of the manuscript.

## Conflict of Interest Statement

FV is member of the Scientific Advisory Board of Biosceptre Ltd., a Biotech involved in the development of P2X7-targeted therapies. The remaining authors declare that the research was conducted in the absence of any commercial or financial relationships that could be construed as a potential conflict of interest.

## Abbreviations

IDO1, indoleamine-2,3-dioxygenase 1; DNR, daunorubicin; AML, acute myeloid leukemia; P2X7R, P2X7 receptor; DCs, dendritic cells; Tregs, T regulatory cells; PB, peripheral blood; ARA-C, cytarabine; mAbs, monoclonal antibodies; 1-MT-l, 1-mthyltryptophano-levo; PD-1, programmed cell death protein 1; p.i., post-inoculum; p/s/cm^2^/s, photons/second/cm^2^/steradian; CTLs, cytotoxic T cells; EM, effector memory; EMRA, effector memory expressing RA; CM, central memory.
