# Supplementary material for: ATP Release from Chemotherapy-Treated Dying Leukemia Cells Elicits an Immune Suppressive Effect by Increasing Regulatory T Cells and Tolerogenic Dendritic Cells
Source: Front Immunol. 2017 Dec 22;8:1918. doi: 10.3389/fimmu.2017.01918 (PMC5744438; doi:10.3389/fimmu.2017.01918)
Supplement: Supplementary file 1 [file Table_1.DOCX]

**Supplementary Tables**

**Table 1S. Patients’ characteristics**

Characteristics of 23 newly diagnosed AML patients undergoing standard “7+3” induction chemotherapy regimen, including continuous infusion of ARA-C at 200 mg/m^2^ for 7 days and DNR at 60 mg/m^2^ for 3 days.

| **UPN** | **Age** | **Sex** | **FAB** | **WBC (cells/μl)** | **Karyotype** | **Genotype** | **Induction chemotherapy regimen** |
| --- | --- | --- | --- | --- | --- | --- | --- |
| 1 | 64 | M | M0-M1 | 148,900 | normal | FLT3^-^, NPM^+^ | “7+3” |
| 2 | 43 | F | M2 | 8,100 | t(8;21) | c-KIT-, FLT3^-^, NPM^-^ | “7+3” |
| 3 | 51 | F | M5 | 77,730 | normal | FLT3 ITD^+^, NPM^+^ | “7+3” |
| 4 | 67 | F | NA | 59,700 | normal | FLT3 ITD^+^, NPM^+^ | “7+3” |
| 5 | 68 | M | M2-M4 | 21,600 | inv(16) | CBF-MYH11 | “7+3” |
| 6 | 57 | M | M1 | 28,600 | normal | FLT3^+^, NPM^-^ | “7+3” |
| 7 | 59 | F | NA | 15,800 | normal | NA | “7+3” |
| 8 | 68 | F | M4 | 4,100 | normal | FLT3^-^, NPM^-^ | “7+3” |
| 9 | 68 | M | NA | 57,000 | normal | FLT3 ITD^+^ | “7+3” |
| 10 | 44 | F | NA | 18,500 | normal | FLT3 ITD^+^ | “7+3” |
| 11 | 58 | F | M2 | 5,200 | normal | NPM^-^, FLT3^-^ | “7+3” |
| 12 | 63 | F | M4 | 25,300 | normal | FLT3^-^, NPM^+^ | “7+3” |
| 13 | 64 | F | NA | 57,400 | normal | FLT3^+^, NPM^+^, DNMT3A^-^, TP53^-^ | “7+3” |
| 14 | 43 | M | M5b | 12,900 | normal | FLT3 ITD^+^, NPM^+^ | “7+3” |
| 15 | 55 | M | M4 | 11,300 | inv(16) | CBF-MYH11^+^, FLT3^-^, NPM^-^, DNMT3A^-^ | “7+3” |
| 16 | 63 | M | M4-M5 | 49,100 | normal | NE | “7+3” |
| 17 | 66 | F | M1-M2 | 30,300 | normal | FLT3^-^, NPM^+^ | “7+3” |
| 18 | 48 | M | M1 | 2,800 | complex | FLT3^-^, NPM^-^, DNMT3A^-^, TP53^-^ | “7+3” |
| 19 | 46 | F | M4 | 95,450 | t(16;16) | FLT3^-^, NPM^-^, TP53^-^, CBFβ-MYH11 | “7+3” |
| 20 | 66 | M | NA | 2,000 | normal | FLT3^-^, NPM^-^ | “7+3” |
| 21 | 19 | M | M0 | 127,600 | t(6;11) | FLT3^-^, NPM^-^, DNMT3A^-^, MLL^-^AF4 | “7+3” |
| 22 | 21 | M | M0-M1 | 8,400 | complex | FLT3^-^, NPM^-^, AML-ETO, RUNX1-RUNX1T1 | “7+3” |
| 23 | 70 | M | M2 | 56,500 | NE | NPM MUT, TP53 WT, WT1 overexpressed | “7+3” |
