# Supplementary material for: ATP Release from Chemotherapy-Treated Dying Leukemia Cells Elicits an Immune Suppressive Effect by Increasing Regulatory T Cells and Tolerogenic Dendritic Cells
Source: Front Immunol. 2017 Dec 22;8:1918. doi: 10.3389/fimmu.2017.01918 (PMC5744438; doi:10.3389/fimmu.2017.01918)
Supplement: Supplementary file 2 [file Data_Sheet_1.DOCX]

**Supplementary methods**

***In vitro* quantification of ATP release**

AML cells were seeded in 96-well flat bottom (1x10^6^/ml) and challenged with chemotherapeutic agents, DNR 500 ng/ml (Sigma) or ARA-C 25 μg/ml (Sigma), for 4 hours. Then, cells were washed and after 20 hours ATP quantification in the supernatants was performed in triplicate using ENLITEN rLuciferase/Luciferin Reagent (Promega), according to manufacturers’ instructions. Luminescence was measured at the single-tube luminometer Glomax 20/20 (Promega), with 10-second RLU (relative light units) signal integration time.

**Flow cytometry on human cells**

Intracellular staining of cytokines and FOXP3 was performed at 4°C for 30 minutes, after cells fixation and permeabilization with 4% paraformaldehyde (VWR) and 0.1% saponin (Sigma). For each sample, isotype-matched irrelevant mAbs staining was used as control. At least 10,000 events were collected from each sample at Gallios Flow Cytometer (Beckman Coulter) or BD Accuri C6 (BD Biosciences). For expanded T cells, data analyses was performed by using FlowJo Single Cell Analysis Software (FlowJo LLC).

**Circulating Tregs staining**

Circulating Tregs in PB of AML patients were assessed by using Human Regulatory T Cell Whole Blood Staining Kit (eBioscience). Briefly, for each sample 100 μl of whole blood was stained with human mAbs CD4 FITC (RPA-T4; eBioscience) and CD25 APC (SK1; eBioscience) at rT in the dark for 15 minutes. After incubation with Lysis Buffer (eBioscience) for 10 minutes in the dark, at rT, cells were fixed and permeabilized by adding 1 ml of Fixation/Permeabilization Buffer working solution (eBioscience), and incubated for 30 minutes at 4°C in the dark. Intracellular staining was performed with anti-human FOXP3 PE (236A/E7; eBioscience), at 4°C for 30 minutes. Isotype-matched irrelevant mAbs were used. At least 10,000 events were collected at BD Accuri C6 (BD Biosciences).

**Western Blot Analysis**

5x10^5^ DCs were lysed at 4°C for 30 minutes in Cell Lysis Buffer (10X), enriched with protease inhibitor Phenylmethanesulfonyl fluoride (PMSF; Cell Signaling Technology). Proteins were quantified with Coomassie G-250 (Thermo Fisher Scientific) by using Bio-photometer (Eppendorf). 40 μg of protein were loaded in Mini-PROTEAN® TGX Stain-Free Precast Gels. Mini-PROTEAN 3 vertical electrophoresis and Trans-Blot turbo instruments for electrophoretic run and blotting from Bio-rad were employed, following manufacturer’s instruction. PBS with 3% of milk, 2% of BSA and 0.5% of Tween was used as blocking solution. For staining we used rabbit anti-human IDO1 Ab (1:20,000, rabbit; courtesy of Dr. METZ, Lankenau Institute for Medical Research, Wynnewood, Pennsylvania) and donkey anti-rabbit secondary Ab (1:20,000) (Santa Cruz Biotechnologies). Goat anti-human Actin Ab (1:5,000) and donkey anti-goat secondary Ab (1:20,000) were used for control staining (Santa Cruz Biotechnologies). Protein bands were detected at Chemidoc instrument (Bio-rad) using Amersham ECL Select WB Detection Reagent (GE Healthcare), following the manufacturer's protocol. Precision Plus Protein Kaleidoscope (Bio-Rad) prestained protein standards were used.

**IDO1 silencing**

IDO1 silencing in DCs was performed by small interfering (si)RNA transfection by using standard nucleofection (Amaxa Nucleofector Tecnology, Lonza) with U02 program. 250 nM non-targeting pool control siRNA and 250 nM IDO1-specific siRNA ON-TARGETplusSMARTpool (Dharmacon) were used . After an overnight incubation, DCs maturation was induced by co-culture with DNR-treated HL-60 cells for 24 hours (ratio 1:1).

**Analyses on WEHI-3B tumors**

Flow cytometry

Tumor infiltrating lymphocytes were purified from tumor mass by Ficoll (GE Healthcare Life Sciences) gradient. For the staining of surface markers, tumor infiltrating lymphocytes were incubated for 30 minutes at 4°C in the dark with the following anti-mouse mAbs: CD45 BV510 (30-F11), CD4 eFluor780 (HL3), PD-1 BV711 (29F.1A12), CD8a eFluor780 (53-6.7), IFNgPerCPCy5.5 (XMG1.2), IL10 BV421 (JES5-I6E3), CD25-BV605 (PC61), Ly6C-APC (HK1.4) and D39-PE (clone DUHA59) according to manufacturer’s instruction. For intracellular staining of Foxp3 APC (FJK-16S) cells were fixed and permeabilized with “Foxp3 / Transcription Factor Staining Buffer Set” (affymetrix eBioscience) then stained for 30 minutes at 4°C in the dark. All mAbs were purchased from affymetrix eBioscience or BD Biosciences. Flow cytometry data were acquired on a LSRFortessa (Becton Dickinson) and analyzed with FlowJo software (version 8.8.7, Tree Star Inc.). For intracellular staining of IDO1 (clone mIDO-48; eBioscience) cells were fixed with paraformaldehyde and permeabilized with saponine buffer (0.5%). For surface DC markers the following mAbs were used: CD11c BV421 (clone HL3; BD Biosciences), MHC-II BV510 (clone M5/114.15.2; BD Biosciences), CD40 APC (clone 3/23; eBioscience) and F4/80 PeCy7 (clone BM8; eBioscience).
