# Supplementary material for: ATP Release from Chemotherapy-Treated Dying Leukemia Cells Elicits an Immune Suppressive Effect by Increasing Regulatory T Cells and Tolerogenic Dendritic Cells
Source: Front Immunol. 2017 Dec 22;8:1918. doi: 10.3389/fimmu.2017.01918 (PMC5744438; doi:10.3389/fimmu.2017.01918)
Supplement: Supplementary file 3 [file Data_Sheet_2.DOCX]

**Supplementary figure legends**

**Figure 1S.** Leukemia-specific IFN-γ production by PB CD4^+^ and CD8^+^ T cells collected before drug administration (PRE) and at different time points after chemotherapy (day +7, +14, +21, +28). Results are expressed as fold change of the percentage of IFN-γ-producing CD4^+^ and CD8^+^ T cells stimulated with autologous AML cells over that observed when T cells were stimulated with autologous non-leukemic CD19^+^ cells. A fold-change of 2 was used as cut-off to define positive (n=15) and negative (n=8) patients.

**Figure 2S.** Characterization of CD8^+^ T cells in 3 AML patients positive for IFN-γ production at day 14 after chemotherapy. (A) FACS analysis of T-cell subsets distribution based on CCR7 and CD45RA cell surface expression (naïve, CM and EM + EMRA). The values are represented as mean ± SEM. (B) Expression of activation markers CD38 and CD28 of stimulated leukemia-reactive CD8^+^ T cells of 3 AML patients at day 14 after chemotherapy by FACS. The values are represented as mean ± SEM; * p < 0.05.

**Figure 3S.** Gating strategy for identification of distinct Tregs subsets. Lymphocytes were gated on a side scatter area (SSC-A) versus forward scatter height (FSC-H) plot to exclude cellular debris and FSC-A vs. FSC-H and then side scatter width (SSC-W) vs. SSC-A plots were used to exclude doublets from the analysis. Live cells were selected in a Vivid Aqua vs FSC-A dot plot. Flow cytometry identification and dissection of CD3^+^CD4^+^CD25^+^CD127^low^ T cells population into CD45RA^+^CD25^+^FoxP3^low^ naïve (Tregs1) and CD15s^+^CD45RA^−^CD25^+^FoxP3^high^ memory Tregs (Tregs2) with high suppressive function and CD45RA^−^CD25^+^CD15s^−^ FoxP3^low^ non-Treg cells (Tregs3) subsets was performed. Representative dot plots are shown.

**Figure 4S.** Post-chemotherapy up-regulation of Ki-67 by circulating Tregs in AML patients. Flow cytometry analysis of the expression of intracellular Ki-67 in Treg1, Treg2 and Treg3 subsets at days 14 and 21 post-chemotherapy. One representative patient is shown (left panel). In the right panel the percentages of proliferating Ki67^+^ cells in Treg1, Treg2 and Treg3 subsets at days 14 and 21 post-chemotherapy are shown. Data are reported as means ± SEM of 2 patients.

**Figure 5S.** Indirect measurement of supernatant ATP levels by quantification of emitted bioluminescence, expressed as relative light unit (RLU) in supernatants of WEHI-3B cells after DNR (500 ng/ml) and ARA-C (25 μg/ml) treatment or untreated (CTR). The values are represented as mean ± SEM of 4 independent experiments; * p < 0.05.

**Figure 6S.** Levels of pro-inflammatory plasma cytokines of leukemia-bearing Balb/cJ mice. Balb/cJ mice were inoculated with WEHI-3B cells and treated with DNR, ARA-C or placebo. IFN-γ, IL-1β, IL-2 and IL-12 were evaluated in plasma samples obtained at post-inoculum day 12. Data are represented as mean ± SEM of 8 mice per condition; * p < 0.05; ** p < 0.01; *** p < 0.001 versus placebo.

**Figure 7S.** FACS analysis of CD11c^low^ and CD11c^high^ myeloid DCs in CD11b^+^ myelomonocytic DCs population after placebo (upper panels), DNR or ARA-C (lower panels) treatment. Representative experiment.

**Figure 8S.** Kinetic measurements of extracellular ATP levels in tumor microenvironment of P2X7 wild type (WT) and knock out (KO) DNR treated mice (n=6), estimated by PmeLUC luminescence.
