# Supplementary figures and images for: ATP Release from Chemotherapy-Treated Dying Leukemia Cells Elicits an Immune Suppressive Effect by Increasing Regulatory T Cells and Tolerogenic Dendritic Cells
Source: Front Immunol. 2017 Dec 22;8:1918. doi: 10.3389/fimmu.2017.01918 (PMC5744438; doi:10.3389/fimmu.2017.01918)

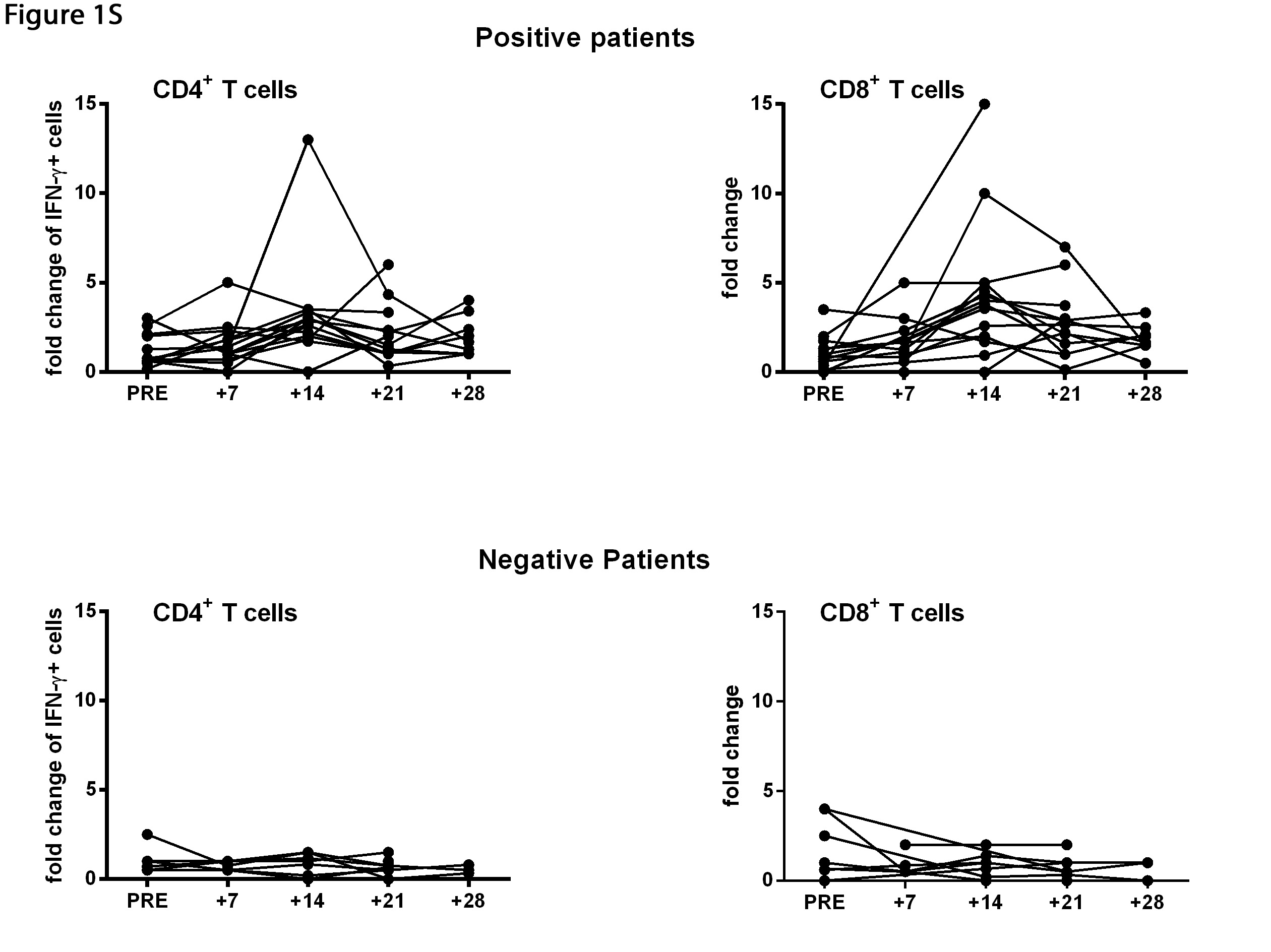

Supplement: Supplementary file 4 [file Image_1.JPEG]

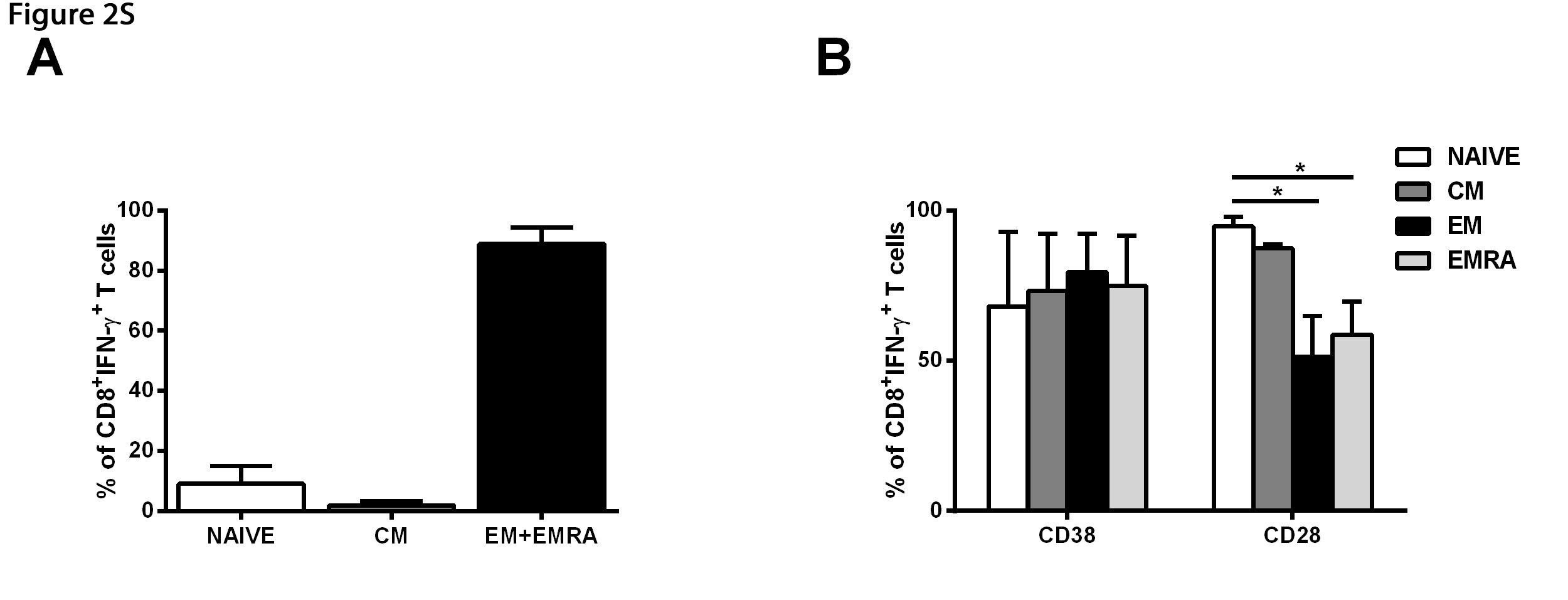

Supplement: Supplementary file 5 [file Image_2.JPEG]

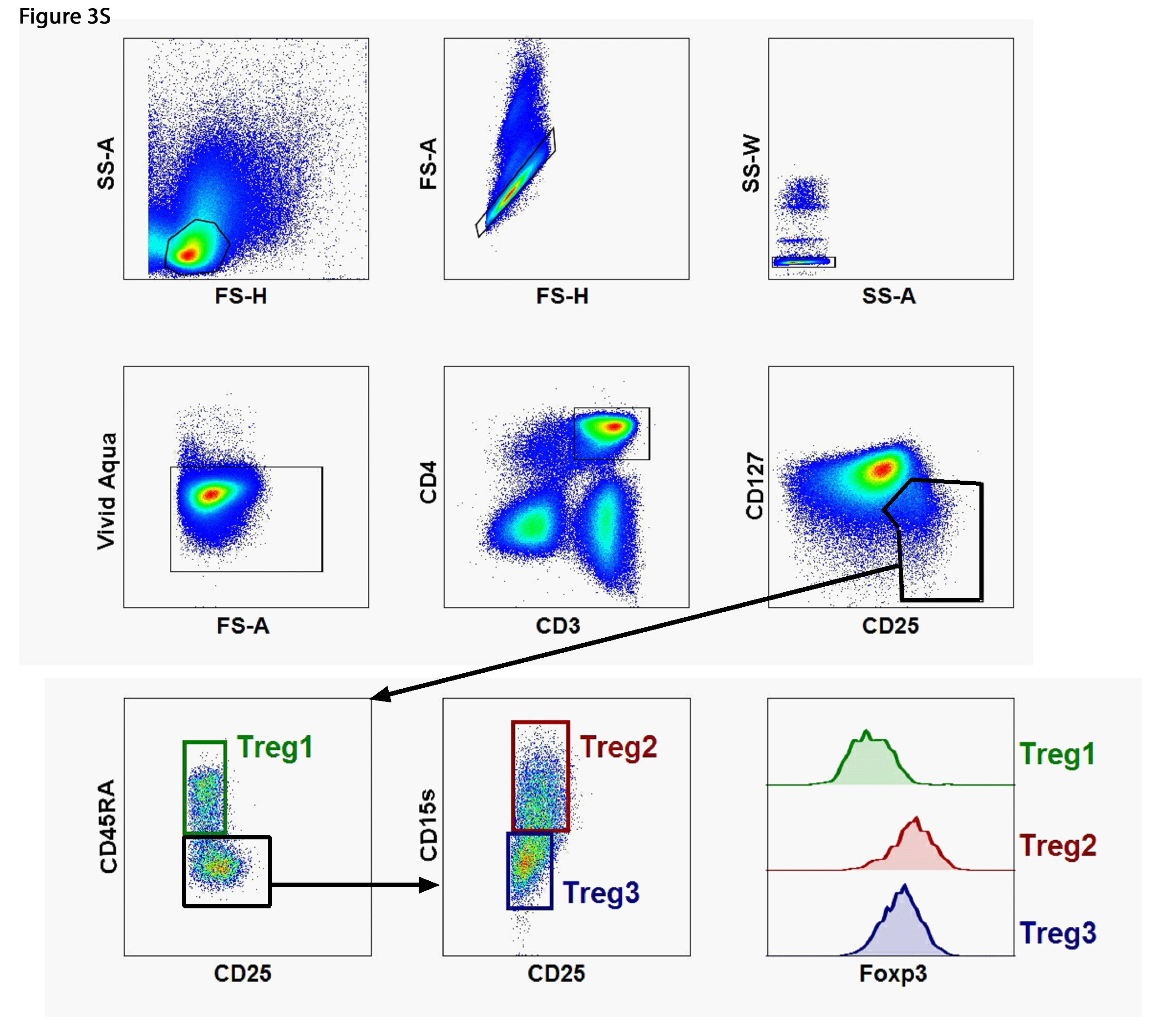

Supplement: Supplementary file 6 [file Image_3.JPEG]

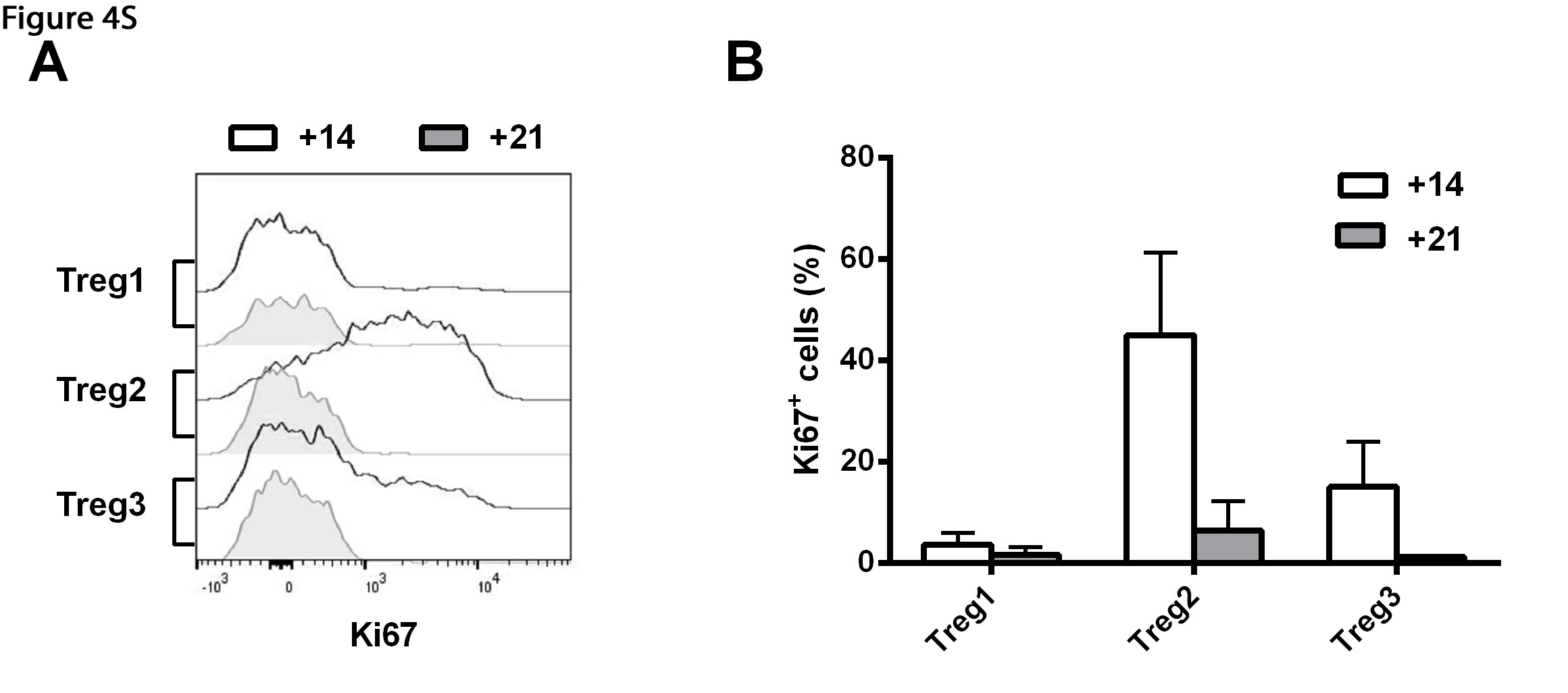

Supplement: Supplementary file 7 [file Image_4.JPEG]

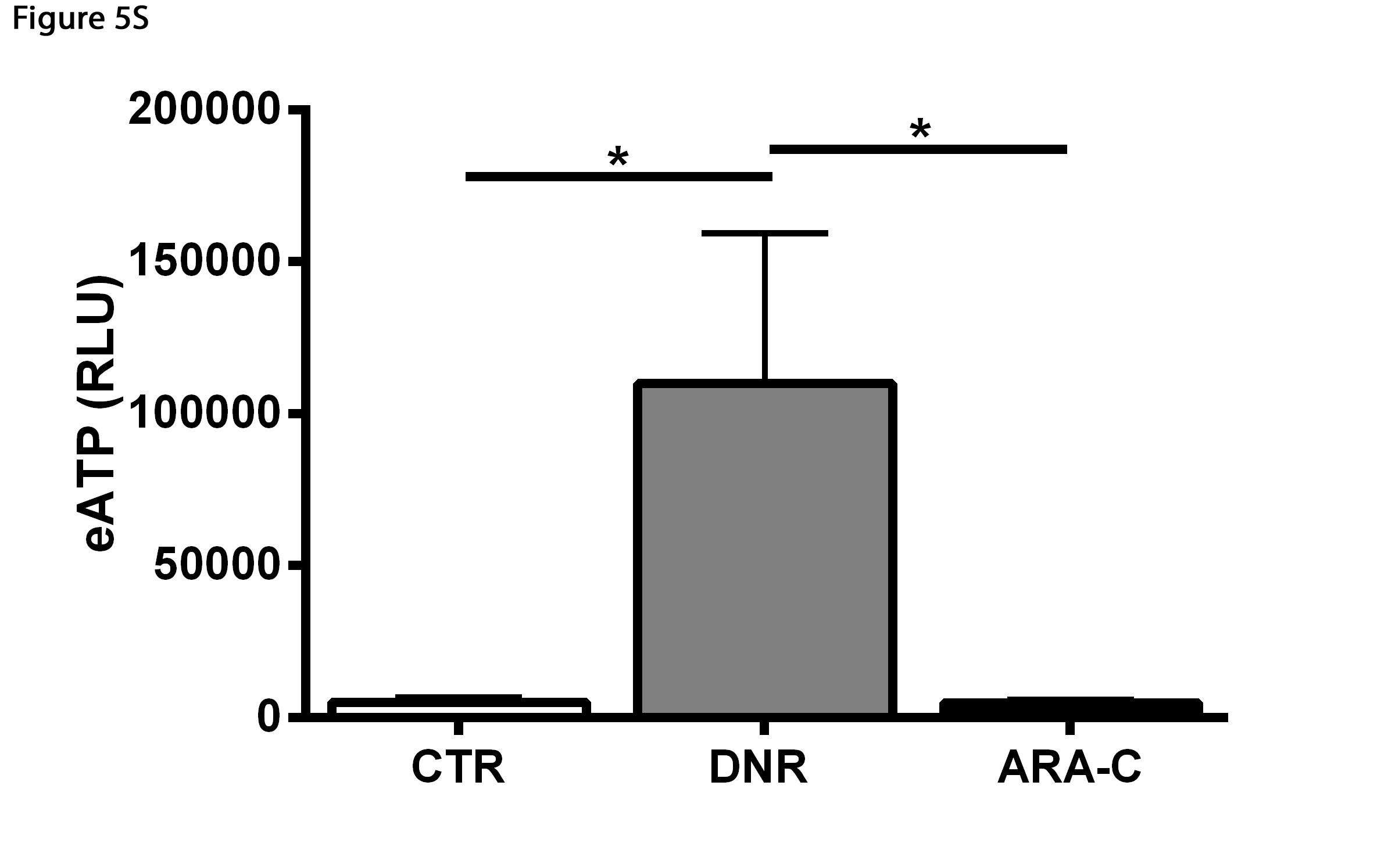

Supplement: Supplementary file 8 [file Image_5.JPEG]

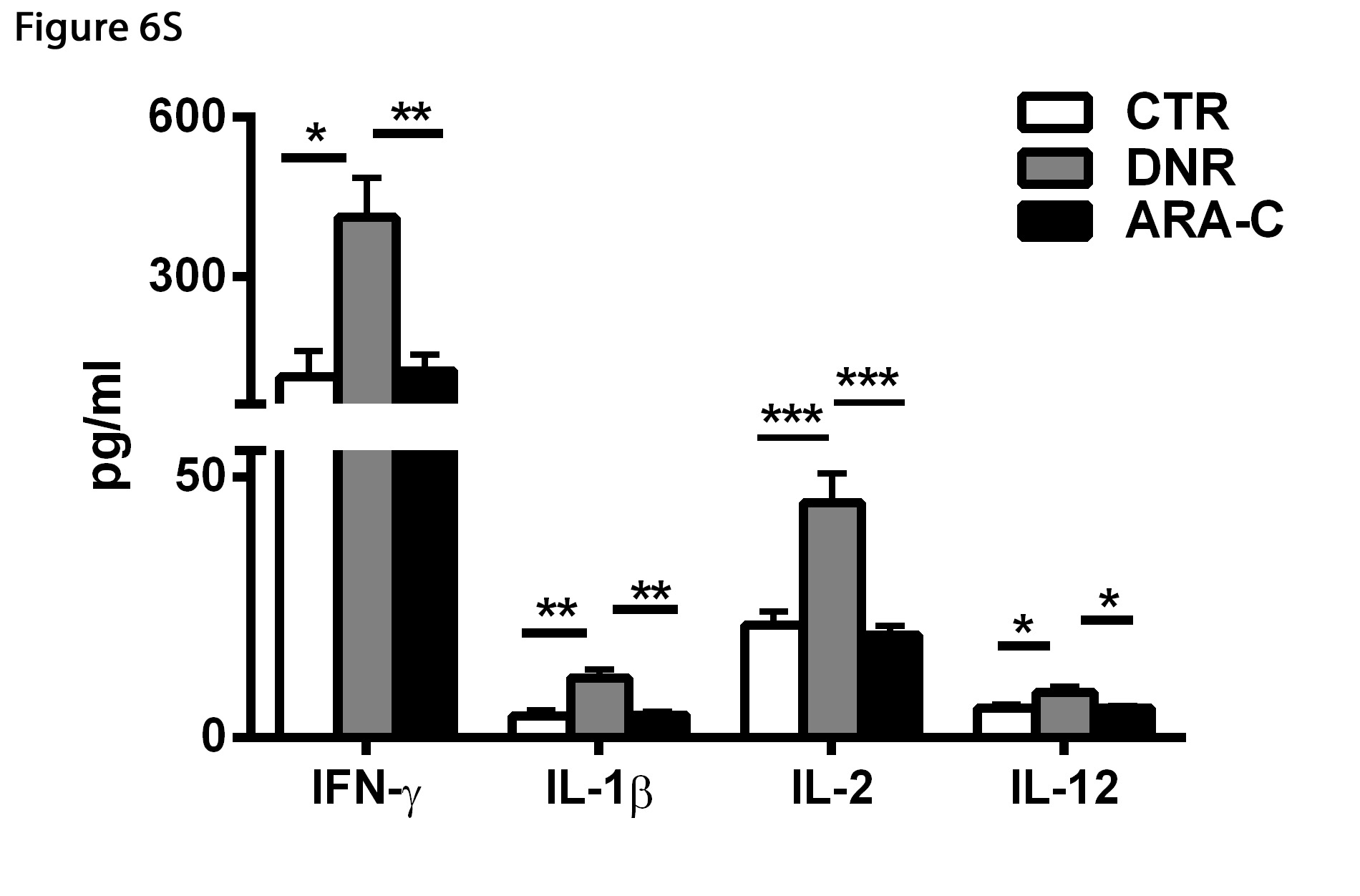

Supplement: Supplementary file 9 [file Image_6.JPEG]

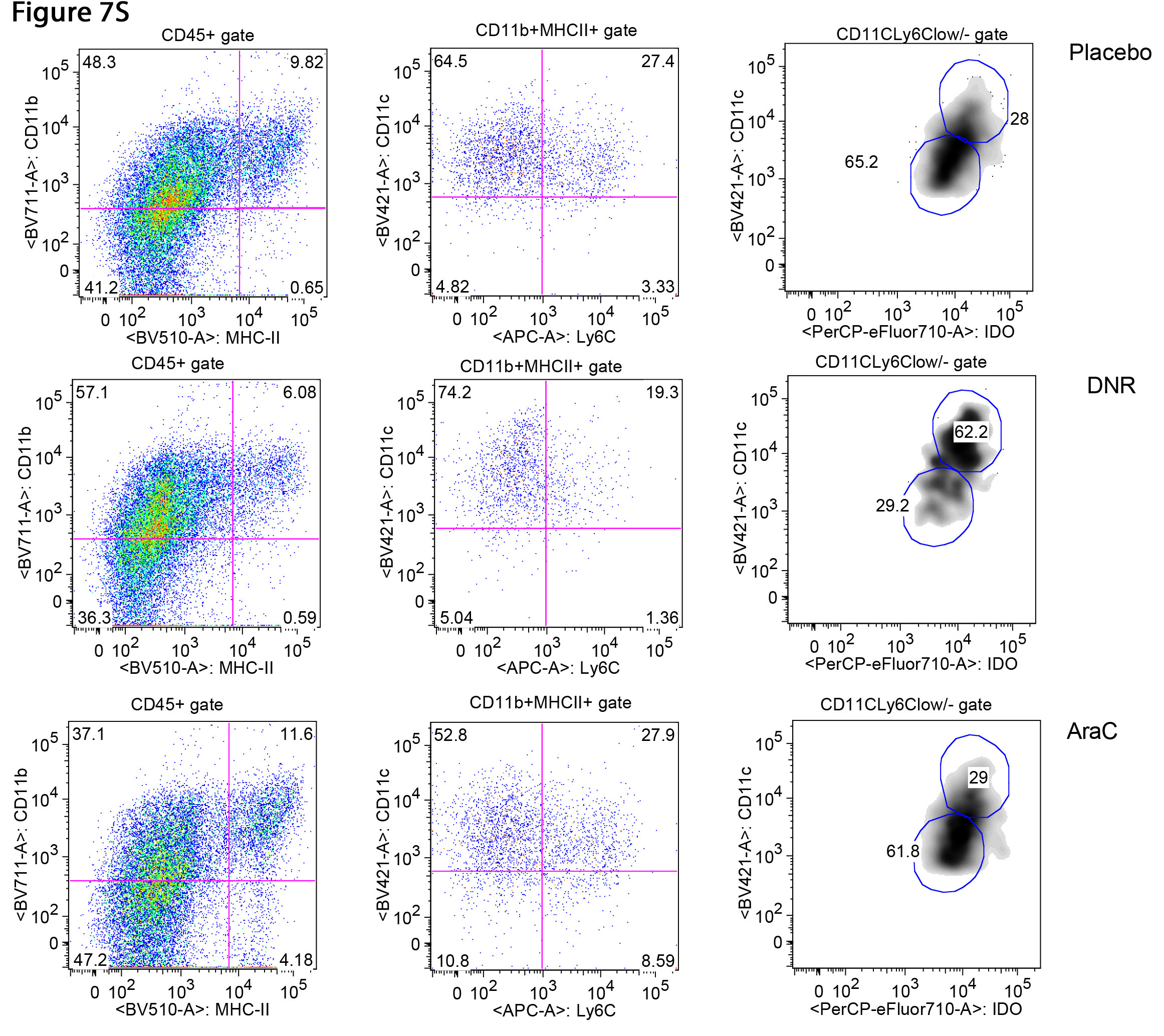

Supplement: Supplementary file 10 [file Image_7.JPEG]

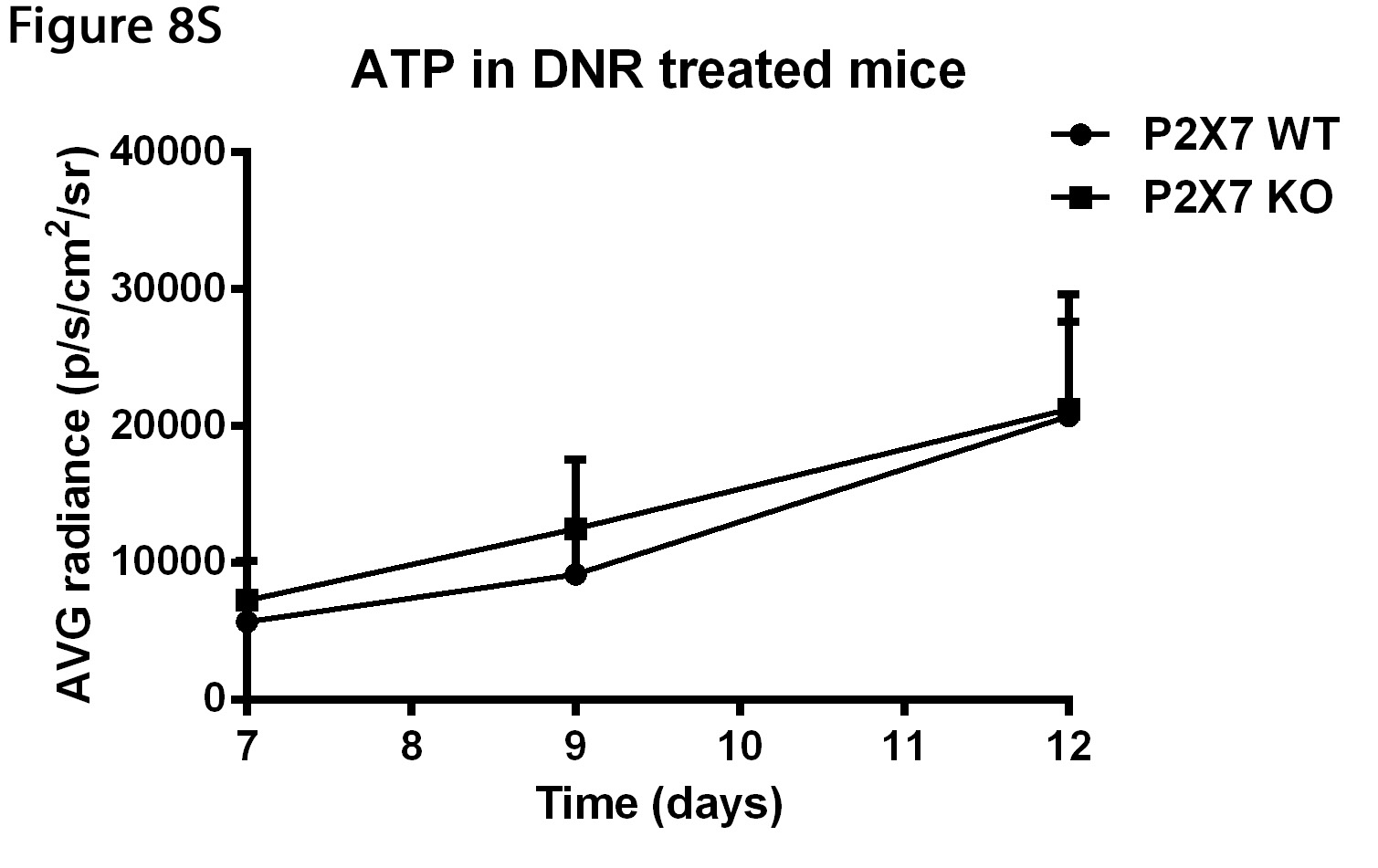

Supplement: Supplementary file 11 [file Image_8.JPEG]

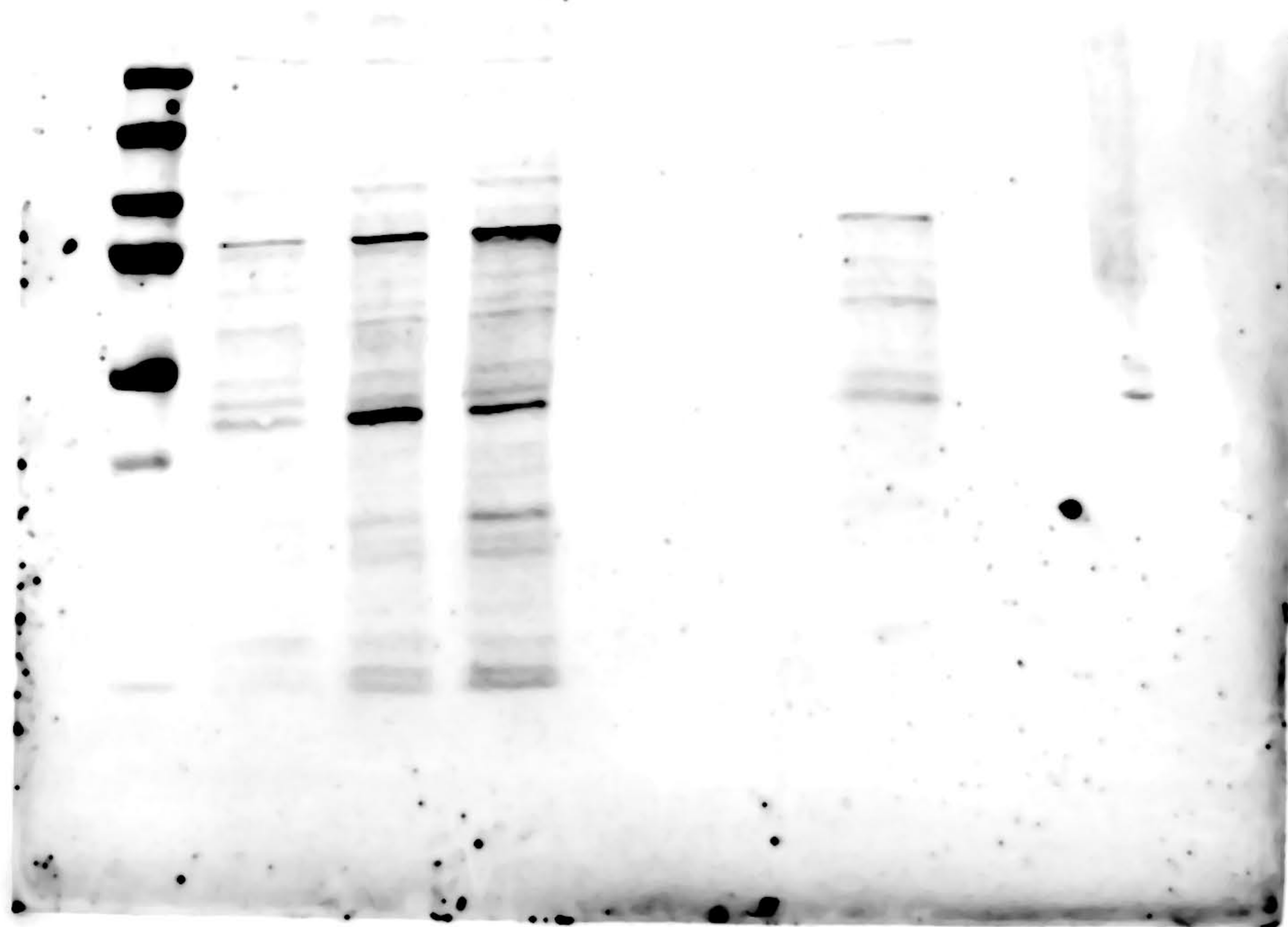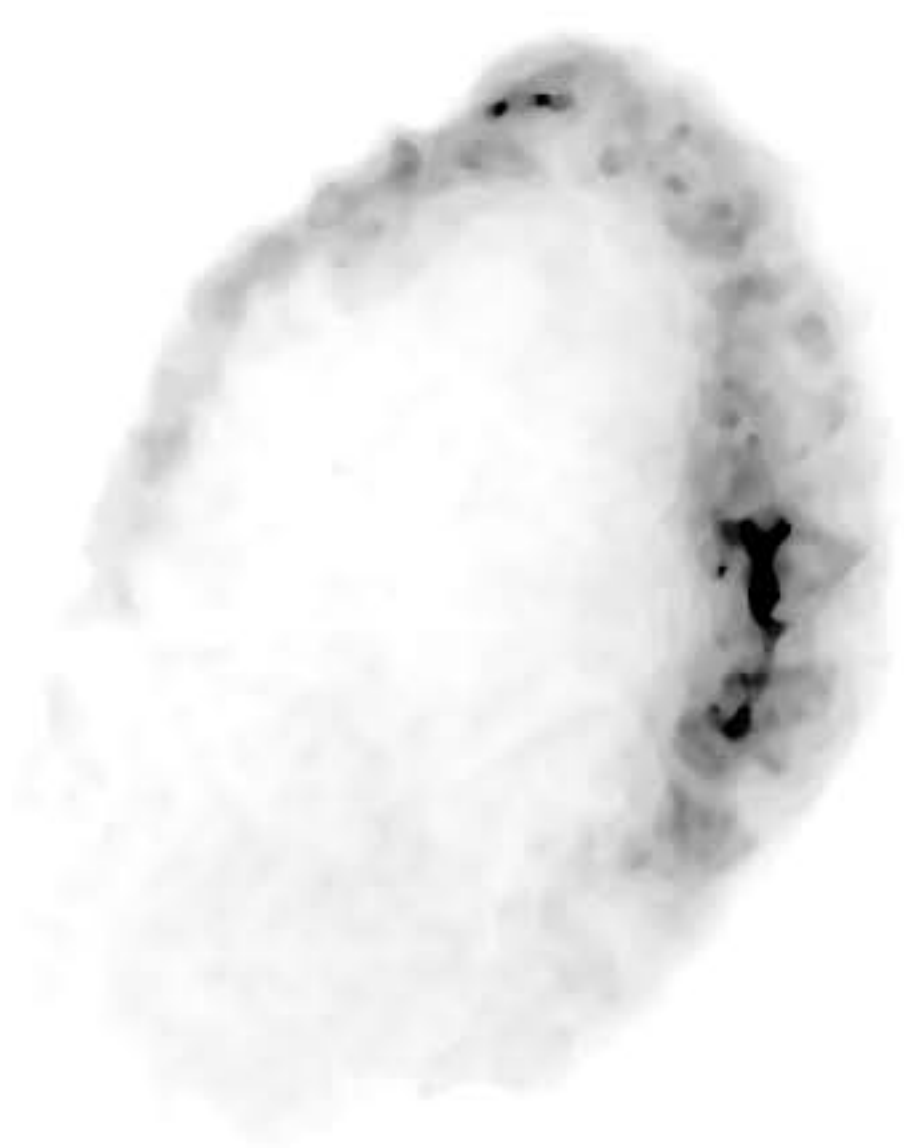

Supplement: Supplementary file 13 [file Image_10.PDF]

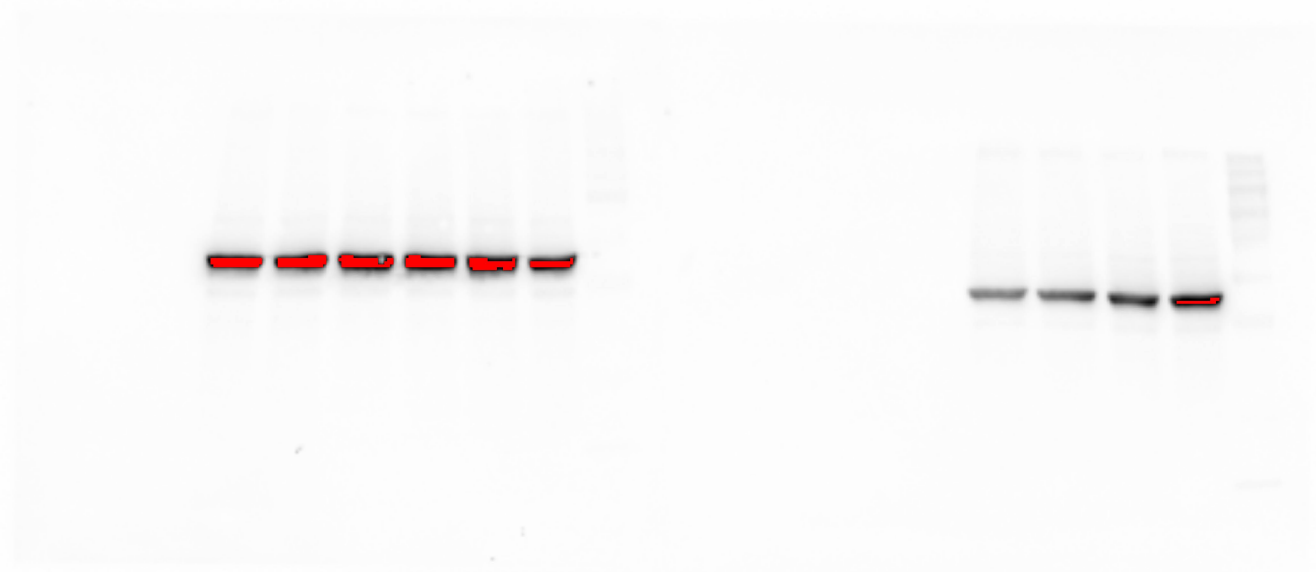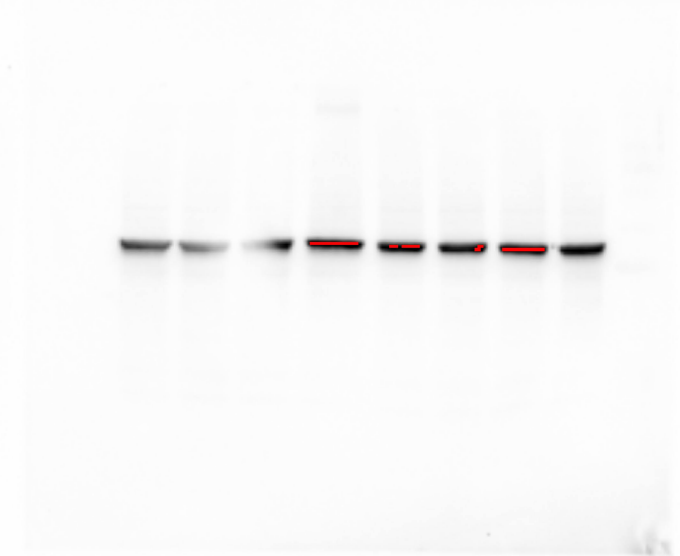

Supplement: Supplementary file 14 [file Image_11.PDF]

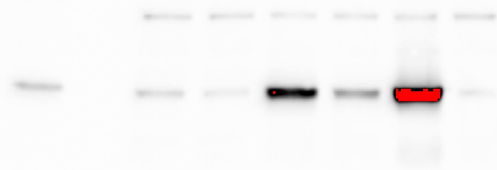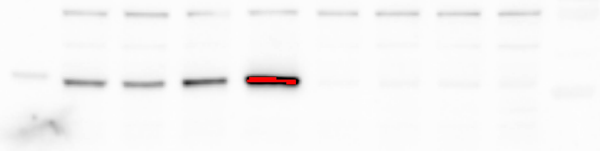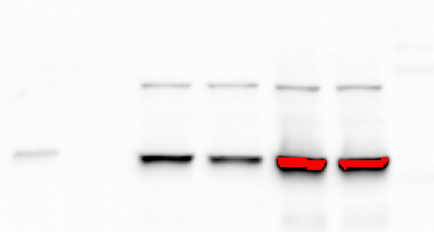

Supplement: Supplementary file 15 [file Image_12.PDF]
