# Supplementary material for: ATP Release from Chemotherapy-Treated Dying Leukemia Cells Elicits an Immune Suppressive Effect by Increasing Regulatory T Cells and Tolerogenic Dendritic Cells
Source: Front Immunol. 2017 Dec 22;8:1918. doi: 10.3389/fimmu.2017.01918 (PMC5744438; doi:10.3389/fimmu.2017.01918)

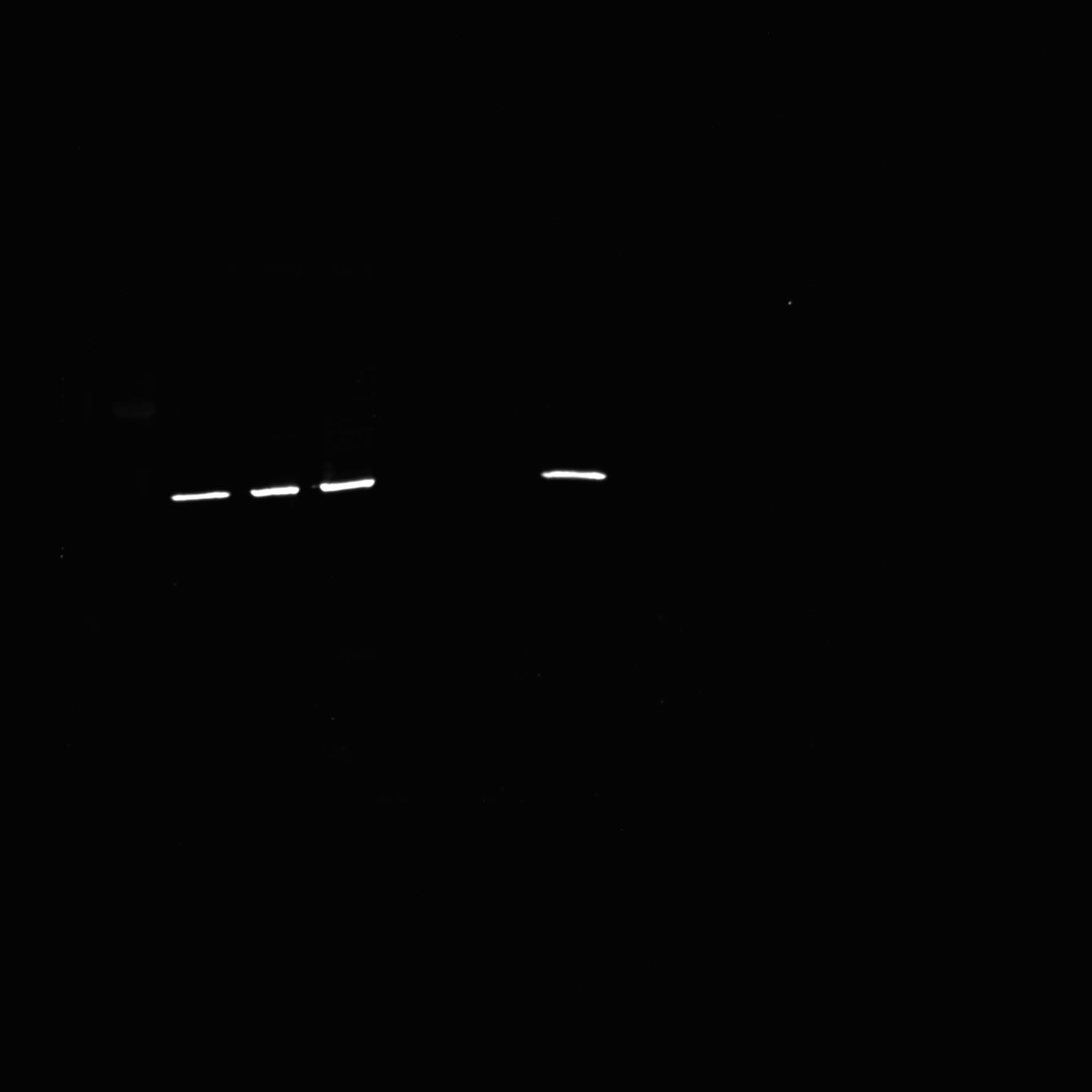

Supplement: Supplementary file 12 [file Image_9.PDF]
